# Trans-radial approach versus trans-femoral approach in patients with acute coronary syndrome undergoing percutaneous coronary intervention: An updated meta-analysis of randomized controlled trials

**DOI:** 10.1371/journal.pone.0266709

**Published:** 2022-04-28

**Authors:** Nagendra Boopathy Senguttuvan, Pothireddy M. K. Reddy, PunatiHari Shankar, Rizwan Suliankatchi Abdulkader, Hanumath Prasad Yallanki, Ashish Kumar, Monil Majmundar, Vadivelu Ramalingam, Ravindran Rajendran, Kesavamoorthy Bhoopalan, Dhamodharan Kaliyamoorthy, Muralidharan T. R., Ankur Kalra, Ramamoorthi Jayaraj, Sivasubramanian Ramakrishnan, Ramesh Daggubati, Sadagopan Thanikachalam, Ashok Seth, Vinay Kumar Bahl

**Affiliations:** 1 Department of Cardiology, Sri Ramachandra Institute of Higher Education and Research (SRIHER), Chennai, Tamil Nadu, India; 2 Adjunct Faculty, Department of Engineering and design, Indian Institute of Technology-Madras, Chennai, India; 3 Department of Medicine, Sri Ramachandra Institute of Higher Education and Research (SRIHER), Chennai, Tamil Nadu, India; 4 Scientist-D, ICMR-National Institute of Epidemiology, Chennai, India; 5 Section of Cardiovascular Research, Heart, Vascular, and Thoracic Department, Cleveland Clinic Akron General, Akron, Ohio; 6 Department of Internal Medicine, New York Medical College, Metropolitan Hospital, New York, New York, United States of America; 7 Department of Cardiology, Velammal Medical College and Hospital, Madurai, India; 8 Department of Cardiology, Apollo Hospitals, Trichy, India; 9 Department of Cardiology, Meenakshi Hospital, Thanjavur, India; 10 Department of Cardiology, Apollo Hospitals, Chennai, India; 11 Department of Cardiovascular Medicine, Heart, Vascular, and Thoracic Institute, Cleveland Clinic, Cleveland, Ohio; 12 UNAA, Darwin, Australia; 13 Department of Cardiology, All India Institute of Medical Sciences, New Delhi, India; 14 Department of Cardiovascular Medicine, WVU Heart and Vascular Institute, Morgantown, India; 15 Department of Cardiology, Fortis Escorts Heart Institute, New Delhi, India; 16 Department of Cardiology, Max- Super-speciality Hospitals, New Delhi, India; University of Messina, ITALY

## Abstract

**Introduction:**

Trans-radial approach (TRA) is recommended over trans-femoral approach (TFA) in patients with acute coronary syndrome (ACS) undergoing percutaneous coronary intervention (PCI). We intended to study the effect of access on all-cause mortality.

**Methods and results:**

We searched PubMed and EMBASE for randomized studies on patients with ACS undergoing PCI. The primary outcome was all-cause mortality at 30-days. The secondary outcomes included in-hospital mortality, major adverse cardiac or cerebrovascular event (MACE) as defined by the study, net adverse clinical event (NACE), non-fatal myocardial infarction, non-fatal stroke, stent thrombosis, study-defined major bleeding, and minor bleeding, vascular complications, hematoma, pseudoaneurysm, non-access site bleeding, need for transfusion, access site cross-over, contrast volume, procedure duration, and hospital stay duration. **We studied 20,122 ACS patients, including 10,037 and 10,085 patients undergoing trans-radial and trans-femoral approaches, respectively**. We found mortality benefit in patients with ACS for the trans-radial approach [(1.7% vs. 2.3%; RR: 0.75; 95% CI: 0.62–0.91; P = 0.004; I2 = 0%). Out of 10,465 patients with STEMI, 5,189 patients had TRA and 5,276 had TFA procedures. A similar benefit was observed in patients with STEMI alone [(2.3% vs. 3.3%; RR: 0.71; 95% CI: 0.56–0.90; P = 0.004; I2 = 0%). We observed reduced MACE, NACE, major bleeding, vascular complications, and pseudoaneurysms. No difference in re-infarction, stroke, and serious bleeding requiring blood transfusions were noted. We noticed a small decrease in contrast volume(ml) {mean difference (95% CI): −4.6 [−8.5 to −0.7]}, small but significantly increase in procedural time {mean difference (95% CI) 1.2 [0.1 to 2.3]}and fluoroscopy time {mean difference (95% CI) 0.8 [0.3 to1.4] min} in the trans-radial group.

**Conclusion:**

TRA has significantly reduced 30-day all-cause mortality among patients undergoing PCI for ACS. TRA should be the preferred vascular access in patients with ACS.

## Introduction

Cardiovascular diseases are one of the leading causes of morbidity and mortality affecting millions of people worldwide [1]. Management of acute coronary syndrome (ACS) has evolved over a period of time to reach its current position. Percutaneous coronary intervention (PCI) is an established treatment of patients with ACS. PCI using trans-femoral approach (TFA) was embraced initially, and was replaced by the trans-radial approach (TRA). Many randomized studies comparing trans-radial and trans-femoral approaches in patients with coronary artery disease are available. Major scientific societies recommend trans-radial procedures in patients with ACS [2, 3]. In contrast to prior studies, a recently published study showed no difference in outcomes according to the access (4). None of the included randomized studies were powered for all-cause mortality events. Hence, we aimed to do an updated systematic review and metanalysis to understand the safety and efficacy of TRA Vs TFA in patients with the ACS undergoing PCI.

## Methods

### Search strategy

We searched PubMed and Embase for all studies on patients with ACS [unstable angina, non-ST elevation myocardial infarction (NSTEMI) and STE myocardial infarction (ST elevation MI)] undergoing PCI (Since inception to April 2021) published in the English language. Also, we looked for cross-references in the screened studies, review articles, and prior similar meta-analysis along with conference proceedings to identify studies that can be potentially included. Our complete search strategy is described in the S1 File.

### Study registration and ethical clearance

Our study protocol is registered with PROSPERO, International prospective register of systematic reviews (CRD42020185367). As it is a meta-analysis of already published studies whose data is available online, Institute ethical committee clearance was not obtained.

### Study eligibility

Only randomized studies on patients with ACS or STEMI undergoing PCI comparing TRA with TFA in the English literature were eligible.

### Eligibility assessment, data extraction, and validity assessment

The Preferred Reporting Items for Systematic Reviews and Meta-Analyses (PRISMA) statement was followed in the execution of this systematic review and meta-analysis. Studies identified through the databases were incorporated into a single database file using Rayyan. Screening of the studies was performed using the title and abstracts after eliminating the duplicates by two independent authors (PR and PS). The full text of the screened articles was methodically assessed later for their eligibility for inclusion into the final data extraction and qualitative data synthesis. Validity assessment for individual studies was performed by two independent authors (PR&PS) using Risk of bias assessment was done using the Cochrane risk-of-bias tool for randomized trials version 2 (RoB 2). We obtained baseline characteristics of patients, procedural details, and clinical outcomes from the included studies. Data were extracted individually by two independent physicians (PR and PS). Any disagreement between the authors in the process of title and abstract screening, full-text screening, data extraction, and validity assessment was resolved by a third author (NS).

### Outcomes

The primary outcome of our study was all-cause mortality at 30 days. The secondary outcomes included in-hospital mortality, major adverse cardio-vascular events (MACE) (composite of death, MI, or stroke) as defined by the study, net adverse clinical event (NACE) (composite of death, MI, stroke, or major bleeding), non-fatal myocardial infarction, non-fatal stroke, stent thrombosis, study-defined major bleeding, and minor bleeding, vascular complication, hematoma, pseudoaneurysm, access site bleeding, non-access site bleeding, need for transfusion, access site cross-over, contrast volume, procedure duration, study defined acute kidney injury, mean difference in creatinine, and hospital stay duration. When an outcome is reported only by a single study, it was not included in the final analysis.

### Statistical analysis

Data extracted from the studies were noted into a Microsoft Excel sheet which was later imported into Review Manager Version 5.3 (The Nordic Cochrane Center, The Cochrane Collaboration Copenhagen, Denmark) and R version 3.6.3 (R Foundation for Statistical Computing, Vienna, Austria) for analysis. **Random effect model was used in our meta-analysis**. We executed a random effects meta-analysis using the Mantel-Haenszel method of pooling risk ratios (RR) with 95% confidence interval (CI) for all outcomes. We computed between-study heterogeneity by using the Higgins I^2^ statistic. We defined low and high heterogeneity as I^2^<25% and >75% respectively. Publication bias for the primary outcome was assessed visually by the asymmetry in funnel plots. We performed a sensitivity analysis using fixed effect model. We intended to do subgroup analysis based on age (age<60/>60), sex, concurrent anticoagulation status, chronic kidney diseases (eGFR<60), Anemia (Hb<9/>9), and diabetes mellitus whenever the data of the same is available in more than two studies. We performed a leave-one-out sensitivity analysis to remove the effect of one study at a time on our results. We also did an analysis restricting to high-quality studies, and studies that included patients with STEMI alone. We calculated the anticipated power of the meta-analysis for major outcomes using information from previous literature and compared them with actual power attained at a 5% significance level. Several candidate covariates (mean age, % of females, % with diabetes, % with hypertension, % of smokers, and % of patients receiving GpIIb/IIIa inhibitors) were examined for association with treatment effect for all-cause mortality in ACS and STEMI patients, separately. A mixed-effects DerSimonian-Laird meta-regression was performed (based on predetermined criteria) to determine factors that significantly affected the treatment effect. Significant covariates were visualized using a bubble plot plotting treatment effect across categories of the covariate. A p-value of <0.05 was considered to be statistically significant. All Analyses were performed utilizing Review Manager version 5.3 (The Nordic Cochrane Center, The Cochrane Collaboration Copenhagen, Denmark) and R version 3.6.3 (R Foundation for Statistical Computing, Vienna, Austria).

## Results

**Fig 1:**
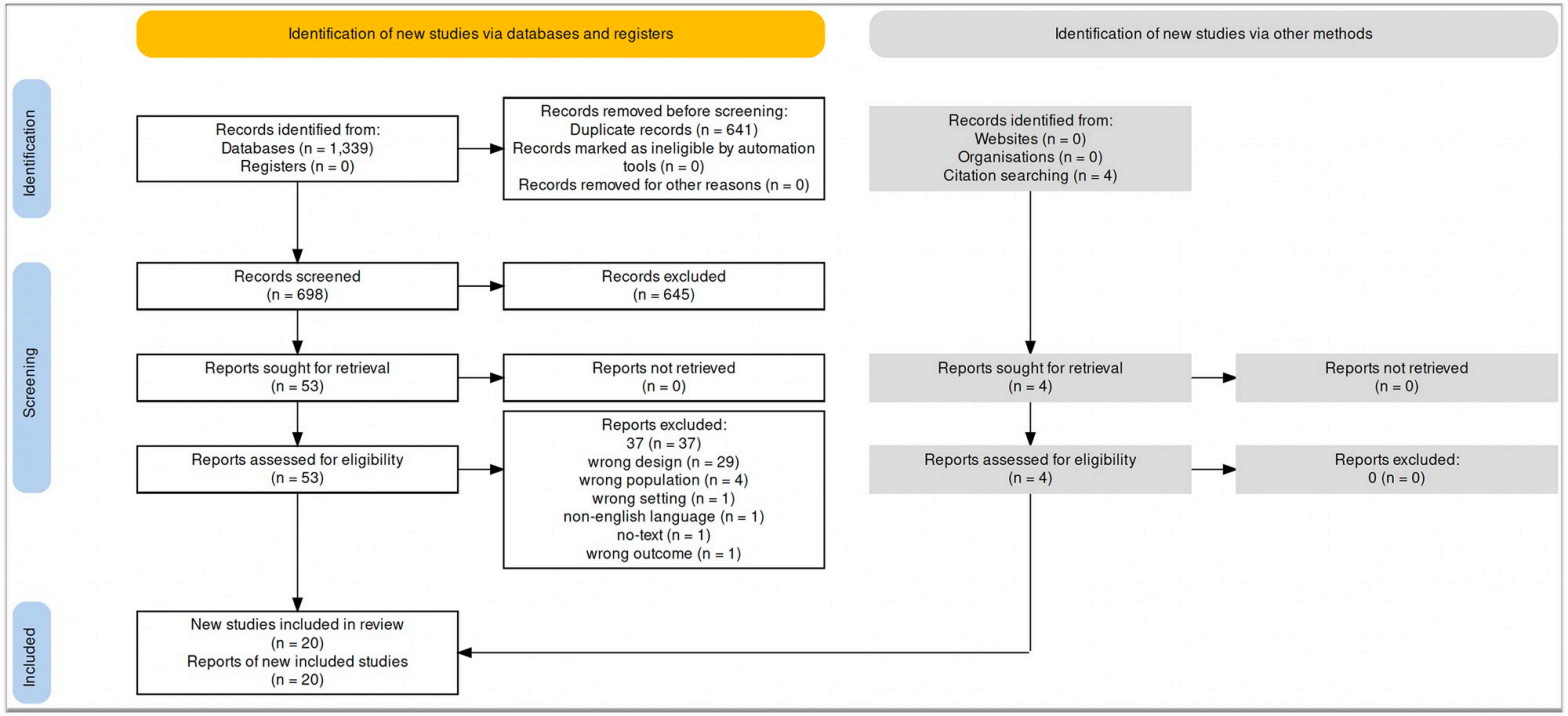
The Preferred Reporting Items for Systematic Reviews and Meta-Analyses (PRISMA) chart. Electronic search from databases and study selection.

Our search strategy resulted in 1,339 studies. After exclusion of duplicates, and inclusion of articles from references, we ended up screening 702 studies for screening. After excluding 645 which were not fulfilling the inclusion criteria, we included 57 articles for full-text screening. We excluded 37 articles due to various reasons as elucidated (Fig 1 & S1 Table in S1 File) that resulted in 20 manuscripts for final quantitative data synthesis with two of those being sub-group analysis published separately.

### Characteristics of included studies including risk-of bias assessment

We included 18 studies for our final analyses [4–21]. Nine of them are single-center studies. Twelve of them were exclusively done in patients with STEMI. Though Jolly et al [13] and Valgimigli et al [19] have conducted their study in patients with ACS, they have reported their outcomes in patients with STEMI separately leading to a total of 14 studies with clinical outcomes for patients with STEMI. Risk of-bias for the primary outcome as assessed by RoB-2 showed a low risk for 5 studies, some concerns for 9 studies, and a high risk for 4 studies (S2 Table in S1 File).

We studied 21,296 patients from 18 studies in our systematic review with 10,616patients in the trans-radial arm and 10,680 patients in the trans-femoral arm. Baseline characteristics of the patient population in the included studies are shown in Table 1. Four of the included studies were having a large patient population (>500 patients) which contributed to the majority of the patient population in our meta-analysis [4, 5, 17, 19]. Trials differed in the use of anticoagulation with unfractionated heparin, low molecular weight heparin and bivalirudin, and usage of vascular-closure devices (Table 1).

**Table 1:** Characteristics of included studies.

**CHARACTERISTICS**	**GAN et al 2009**	**HOU et al 2010**	**TEMPURA** **(SAITO et al)**	**MATRIX** **(VALGIMIGLI et al)**	**YAN et al 2008**	**WANG et al 2012**
**Trial design**	RCT; MC June 2004- July 2007	RCT; SC Aug 2005- Sept 2008	RCT; SC July 1999- Feb 2001	RCT; MC Oct-11-2011- Nov-7-2014	RCT; MC June 2005- June 2007	RCT; SC July 2008- Dec 2010
**Population**	STEMI; 195 PTS	ACUTE MI; 200 PTS	STEMI; 149 PTS	ACS; TR- 4197; TF-4207	STEMI; TR- 57, TF-46	STEMI; TR- 60, TF- 59
**Age**	53.6±12.5/52.3±11.9	64.9±8.4/66.2±7.7	66±12/67±10	65.6(11.8)/65.9(11.8)	70.3±7.5/71.4±8.4	59.8±12.4/60.2±11.4
**Mean guiding catheter per patient (mean) sd**	-	-	1.1±0.4/ 1.1±0.3			
**Inclusion criteria**	Typical chest pain >30min and <12 houres; nitrate losing efficacy, with ST segment elevation >0.1mV in the limb leads or >**0**.2mV in 2 or more adjacent chest leads.	Patients with AMI	**Pa**tients with AMI-STEMI within 12 hr from onset	Patients with high-risk unstable angina, NSTE-ACS, or STEMI undergoing invasive approach	Chest pain for more than 30 minutes without response to nitroglycerine, ST elevation >1mm in 2 or more contiguous leads.	Typical Clinical presentation, ST elevation of over 0.2mm, Received Intravenous thrombolysis within 6 hours from symptom onset in non-PCI hospital, Admitted to hospital within 12 hours after Intravenous thrombolysis.
**Exclusion criteria**	Negative Allen’s test	Femoral approach due to Cardiogenic shock, History of CABG Negative Allen test Non palpable radial artery	Radial artery pulse was too weak for successful radial artery puncture. If the culprit vessel was the previous coronary bypass graft and if the operator for that particular patient did not consider that both TRI and TFI would be equally feasible.	Stable or silent coronary artery disease; LMWH in the previous 6 h; glycoprotein IIb/IIIa inhibitors in the previous 3 d; any PCI in the previous 30 d; contraindications to angiography, including but not limited to severe peripheral vascular disease	Cardiogenic shock, Non-palpable radial artery, Negative Allen test, Chronic renal failure	Contraindications of thrombolysis, History of CABG, Cardiogenic shock, Known difficulties with Femoral or radial approach, Pathologic Allen’s test Pre-procedural implantation of transient pacemaker or IABP, Chronic renal insufficiency with potential necessity of using radial artery as native fistula, Hemodialysis patients with AV fistula Patient refusal.
**Minimum expertise required yes/No**	-	> 200 cases of TRI		>75 transradial coronary procedures within previous year	Those who performed >500 cases of TRI.	Those who performed >500 cases of TRI.
**Primary outcome of the study**	-		MACE during the initial hospitalization period and the 9-month follow-up period.	MACE: death, nonfatal MI, and stroke; NACE: non–CABGrelated major bleeding, BARC type 3 or 5 or MACE	-	-
**Anticoagulant used**	-	-	-	Before Cathlab- UFH- 1239 VS 1236 Bivalirudin- 4 VS 2 LMWH- 684(16.3%) VS 738(17.5%) In-Cathlab- UFH- 2094(49.9%) VS 1916(45.5%) Bivalirudin- 1683(40.1%) VS 1712(40.7%) LMWH- NA	-	-
**Vascular closure device**	-	-	-	-	-	-
**GpIIb/IIIa inhibitor**	28(31.1%) VS 36 (34.3%)	28 (28%) VS 20 (20%)	Not Approved for Use In Japan then	Before Cath Lab—8(0.2%) VS 7(0.2%) In-Cath Lab-574(13.7%) VS 520(12.4%)	-	33(55%) VS 30 (58%)
**Chronic kidney disease (as defined by the study)**	-	-	-	Renal failure and dialysis **TR-46+4 (1.2%)** **TF-63 (1.5%)**		GIVEN LAB VALUES (micromol/L); SCr before PCI- TR- 83±14.9 TF- 76.1±27.1 SCr 72 hours after PCI- TR- 93.7±20.2 TF- 86.1±19.3
**CHARACTERISTICS**	**RIVAL** **(JOLLY et al 2011)**	**ETRIBY et al 2017**	**MANN et al 1998**	**RADIAL-AMI** **(CANTOR et al)**	**OCEAN RACE** **(KOLTOWSKI et al)**	**LI et al 2007**
**Trial design**	RCT; MC June-6-2006-Nov-3-2010	RCT; SC Dec-13- Dec ‘15	RCT; SC April-July 97	RCT; MC	RCT; SC OPEN LABEL Sept 2010- Oct 2012	RCT; SC June 2004- June 2006
**Population**	ACS- TR-3507, TF- 3514	ACS- 100 PTS	ACS; 142 PTS	STEMI, 50 PTS	STEMI, TR-52, TF-51	AMI; TR-184, TF-186
**AGE**	62±12/62±12	55.18±8.1/55.94±8.76	63/62	52(48,60)(MEDIAN)/ 58(49,72)	61(49.7–72.2)/62.8(50.2–75.4)	56.5±10.9/55.4±12.8
**Mean guiding catheter per patient (mean) sd**						
**Inclusion criteria**	Patients with ACS, with or without ST-segment elevation, and planned invasive approach	Recent onset acute coronary syndrome (whether (UA)/(NSTEMI)(STEMI)) undergoing revascularization via PCI.		All patients with STEMI for primary and rescue PCI, patients could be enrolled within 12 hours of symptom onset and within 12 hours of thrombolysis, respectively.	Patients with STEMI, symptoms between 20min and 24 h of symptom onset, undergoing PCI	Patients admitted as Acute MI(AMI)
**Exclusion criteria**	Cardiogenic shock, severe peripheral vascular disease precluding a femoral approach, or previous CABG with use of >1 internal mammary artery	Cardiogenic shock or resuscitated from cardiac arrest, history of CABG or chronic kidney disease.		Patients in cardiogenic shock, abnormal Allen’s test result, or had contraindications to GP IIb/IIIa inhibitor use (active bleeding, major surgery/biopsy/significant trauma in the past 6 weeks, SBP >200mmHg or DBP>110 mm Hg, INR >2, recent noncompressible vascular puncture, central nervous system structural damage or stroke/ transient ischemic attack within the last 6 months, baseline platelet count <100000 cells/AL).	INR > 1.4 Thrombocytopenia < 100 × 103 Previous CABG Known vascular access difficulties or complications Active bleeding Gastric or duodenal peptic ulcer Current or planned dialysis Severe liver failure (MELD > 10 points) Uncontrolled hypertension (> 160/100 mm Hg) Cardiogenic shock Low compliance to long-term follow-up	Negative Allen test Aorto-arteritis Cardiogenic shock Non-palpable radial artery Severe tortousity of radial arteries Body height <150cm
**Minimum expertise required yes/no**	>50 transradial coronary procedures within previous year			All operators in this study had performed >100. transradial PCI procedures before the study.	Procedures were performed by independent radial operators who carry out at least 200 PCIs per year using a radial approach, and operators who were in training (< 200 PCI per year).	-
**Primary outcome**	NACE: Death, MI, stroke, or non–CABG-related major bleeding	-		The primary efficacy end point of the trials was reperfusion time (time from local anesthesia infiltration to the first balloon inflation). The primary safety end points of the trial were major bleeding (intracranial or retroperitoneal bleeding, a drop in hemoglobin level >5 g/dL or hematocrit>15%, or whole blood or packed red cell transfusions) and access site complications (hematoma >5 cm, pseudoaneurysm, arteriovenous fistula, access site rebleeding after initial hemostasis) during the initial hospitalization.	The primary endpoints were major bleeding by the REPLACE-2 scale and minor bleeding by the EASY scale (TR arm) or the FEMORAL scale (TF arm).	-
**Anti-coagulation used**	UFH- 1168(33.3%) VS 1110(31.6%) Bivalirudin- 76 (2.2%) VS 109 (3.1%)	-	-	-	-	UFH
**Vascular closure device**	-	-	-	0 VS 2(8%)	-	-
**GP IIb/IIIa inhibitor**	887 (25.3%) VS 844(24%)	2 (4%) VS 9 (18%)	10(15%) VS 8(10%)	24(95%) VS 23(92%)	31 (59.2%) VS 34 (66.7%)	-
**Chronic kidney disease (as defined by the study)**					TR- 6 (12%) TF- 9 (18.4%)	
**CHARACTERISTICS**	**FARMI- (BRASSELET et al)**	**RADIAMI- (CHODOR et al2009)**	**RADIAMI-II** **(CHODOR et al 2011)**	**SAFARI STEMI** **(LE MAY et al 2020)**	**RIFLE- STEACS** **(ROMAGNOLI et al)**	**STEMI RADIAL** **(BERNAT et al)**
**Trial Design And Centre**	RCT;MC Jan 2004-Sept 05	RCT; SC April 2005- June 2006	RCT; SC Nov 2006- March 2008	RCT; MC July 2011- Dec 2018	RCT; MC Jan 2009- July 2011	RCT; MC Oct 2009-Feb 2012
**Population**	STEMI; 114 PTS	STEMI 100 PTS	STEMI; 108 PTS	STEMI; 2292 PTS	STEMI; 1001 PTS	STEMI; 707 PTS
**Age**	60±12/58±13	59.9±9.4/59.1±9.0	62.1±9.3/57.6±10.3	61.6±12.3/62.0±12.1	65(56–75)/ 65(55–77)	60±12/58±13
**Mean guiding catheter per patient (mean) sd**	1.24±0.68/ 1.11±0.42					
**Inclusion criteria**	Acute coronary syndrome with ST segment elevation associated with sustained chest pain, undergoing PCI	Age between 18 and 75 years; Presence of MI- ST elevation defined as retrosternal pain lasting longer than 20 minutes, but not longer than 12 hours,	ACS with ST segment elevation associated with retrosternal pain lasting between 20 min and 12 h, undergoing PCI	Patients with STEMI who were referred for primary PCI within 12 hours after symptom onset	Patients suspected of having STEMI planned for early revascularization strategy, within 24 h of symptom onset	Patients with STEMI, within 12 h of symptom onset, undergoing PCI
**Exclusion criteria**	Haemodynamic instability (ie, Killip state .2 or cardiogenic shock), the need for an intra-aortic balloon pump or temporary pacemaker, a history of a coronary artery bypass graft (CABG) or intolerance to abciximab	Age over 75 years; Killip class III or IV; Necessity of an intra-aortic balloon pumping placement before the CA; Necessity of an endocavitary stimulating electrode placement before the CA; Height < 150 cm; history of coronary artery by pass grafting (CABG), if the infarction may be due to a closed venous or arterial bypass graft	Killip class III or IV. Necessity to use an intra-aortic counterpulsation balloon or temporary right ventricular pacing, with the decision made before coronary arteriography (CA). Patient’s height < 150 cm. History of coronary artery bypass grafting (CABG).	Patients who had received fibrinolytic therapy, had been prescribed oral anticoagulant therapy, orhadundergoneprevious coronary arterybypass graft (CABG) surgery.	Contraindication to radial or femoral vascular access (abnormal result on Allen test, severe peripheral vascular disease), recent stroke (within 4 wk), oral anticoagulation, or other severe bleeding diathesis	Cardiogenic shock, prior aortobifemoral bypass, absence of bilateral radial or femoral artery pulses, negative result on Allen test or Barbeau test type D curve, oral anticoagulation
**Minimum expertise required yes/No**		Many years’ experience of heart catheterization with TFA (300–400 PCI per year), who had performed at least 50–100 interventions using TRA	Three physicians with 17–20 years of experience in performing PCI via TFA and several years experience in performing PCI via TRA, took part in the study.	Operators typically performed more than 250 PCI procedures annually.	>150 PCIs/y with adequate expertise in both approaches, minimal proficiency criteria of >50% transradial coronary procedures per year	>200 PCIs/y in high-volume radial centers (>80% cases/y)
**Primary Outcome**		-	-	All-cause 30-day mortality	NACE: Cardiac death, MI, stroke, TLR, and non–CABG-related per protocol bleeding	Major bleeding and vascular access-site complications requiring intervention
**Anticoagulation Used**	-	-	-	UFH- 135(11.9%) VS 88(7.6%) BIVALIRUDIN- 1001 (88.1%) VS 1068 (92.4%)	UFH- DOSE BIVALIRUDIN- 40 (8%) VS 36 (7.2%)	LMWH- 6(1.7%) VS 0 UFH- DOSE GIVEN (NOT THE POPULATION)
**Vascular Closure Device Used**	-	-	-	63(5.5%) VS 789 (68.3%)	-	4(1.1%) VS 136(38%)
**Gp IIb/IIIa inhibitor**	-	22(44%) VS 21(42%) Abciximab	25 (51%) VS 32(54%) Abcixmab	69(6.1%) VS 68(5.9%)	337 (67.4%) VS 350(69.9%)	155(45%) VS 162 (45%)
**Chronic kidney disease (as defined by the study)**				eGFR at baseline (mL/min) TR- 101.7 TF- 103.8		

**Abbreviations**: TR- Trans-radial; TF-Transfemoral; UFH- unfractionated heparin; LMWH- Low molecular weight heparin; ACS- Acute coronary syndrome; STEMI- ST elevation myocardial infarction.

### Clinical outcomes

**Fig 2:**
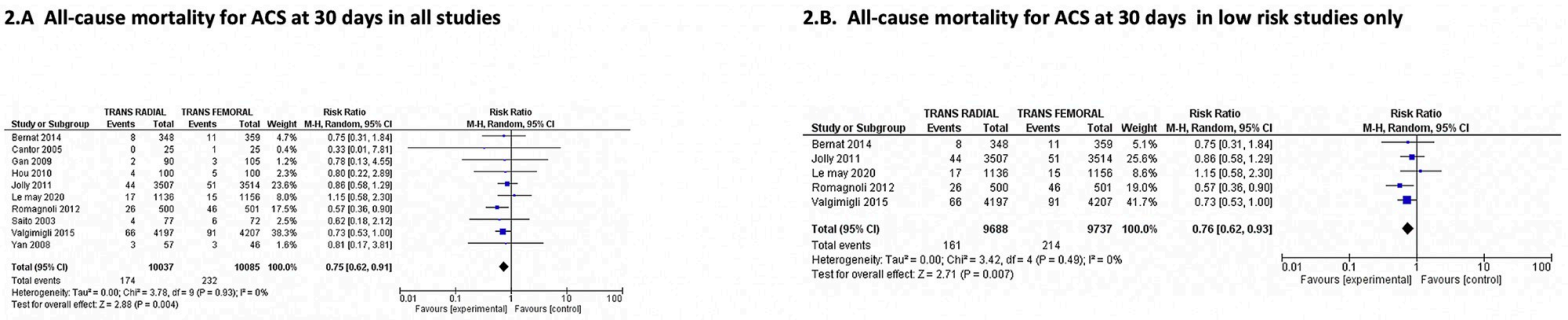
Comparison of Trans-radial approach (TRA) versus Trans-femoral approach (TFA) in patients with acute coronary syndrome showing that TRA is associated with reduced risk of all-cause mortality at 30 days (2.A) in all studies and in studies with low-risk bias (2.B). (B). ACS = Acute coronary syndrome; M-H = Mantel-Haenszel; CI- = confidence interval.

The primary outcome of the study being 30-day mortality in patients with ACS was available in 10 studies only. Out of 20,122 patients with 10,037 patients in the trans-radial arm and 10,085 patients in the trans-femoral arm, we observed 30-day mortality in 174 and 232 patients, respectively underscoring the mortality benefit with the trans-radial approach [(1.7% vs. 2.3%; RR: 0.75; 95% CI: 0.62–0.91; P = 0.004; I^2^ = 0%); Fig 2a]. A funnel plot observation showed no publication bias (S1 Fig in S1 File). A sensitivity analysis using a fixed-effect model revealed the same results (S3 Table in S1 File). A leave-one study at a time analysis showed a similar result favoring trans-radial procedure. When we restricted our analysis to high-quality RCTs with low-risk bias, we found similar results [(1.7% vs.2.2%; RR: 0.76; 95% CI: 0.62–0.93; P = 0.007; I^2^ = 0%) Fig 2b]. There was no significant difference in the primary outcome when subgroup analysis was done based on the use of bivalirudin as the predominant anticoagulant. (S2 Fig in S1 File) Similarly, subgroup analysis based on clinical presentation i, e., NSTEMI Vs STEMI showed no effect on the primary outcome (S3 Fig in S1 File).

**Fig 3:**
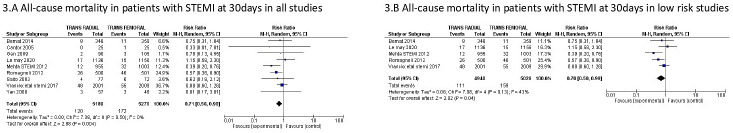
Comparison of Trans-radial approach (TRA) versus Trans-femoral approach (TFA) in patients with STEMI showing that TRA is associated with reduced risk of all-cause mortality at 30 days (3.A) in all studies and in studies with low-risk bias (3.B) (B). STEMI = ST elevation myocardial infarction; M-H = Mantel-Haenszel; CI- = confidence interval.

We performed a separate analysis for studies with clinical outcomes for patients with STEMI. Out of 14 studies with a patient population of 11,027, only 9 studies with a total population of 10,465 patients reported 30- day mortality. We observed 120 events in the trans-radial arm and 172 events in the trans-femoral arm that resulted in a significant mortality benefit with trans-radial procedures as compared with trans-femoral procedures in patients with STEMI [(2.3% vs. 3.3%; RR: 0.71; 95% CI: 0.56–0.90; P = 0.004; I^2^ = 0%) Fig 3a]. When we restricted our analysis to studies with low-risk bias, we found a similar result with reduced 30-day mortality in trans-radial arm [(2.2% vs. 3.1%; RR: 0.70; 95% CI: 0.50–0.99; P = 0.04; I^2^ = 43%); Fig 3b].

In patients with ACS, we found reduced MACE with trans-radial procedures [(5.8% versus 6.7%; RR: 0.87; 95% CI: 0.78–0.97; P = 0.009; I^2^ = 0%; S4 Fig in S1 File]. However, when we restricted our analysis to high quality studies only, we observed no difference between the two arms [5.9% Vs 6.7%; RR: 0.89; 95% CI: 0.76–1.03; P = 0.11; I^2^ = 24%; S5 Fig in S1 File]. When we performed sub-group analysis on patients with STEMI only, we found reduced MACE with trans-radial procedures [(4.8% versus 5.6%; RR: 0.82; 95% CI: 0.68–1.00; P = 0.05; I^2^ = 14%); S6 Fig in S1 File]. However, we observed no difference in MACE when restricted to high quality studies with STEMI patients alone [(4.8% VS 5.6%; RR: 0.83; 95% CI: 0.64–1.07; P = 0.15; I^2^ = 47%) S7 Fig in S1 File].

### Power of the meta-analysis for various outcomes and meta-regression

For the primary outcome, all-cause mortality at 30 days in patients with ACS, both anticipated and actual power was very high (>90%) regardless of the quality of studies (S8A-S8D Fig in S1 File). For MACE at 30 days, the actual power was much higher (>95%) compared to the anticipated power of <45%, regardless of the quality of studies (S8E-S8H Fig in S1 File). In patients with STEMI, a similar pattern was observed with >99% power for all-cause mortality at 30 days, regardless of the quality of studies included (S8I-S8K Fig in S1 File) For MACE among STEMI, the actual power for all included studies (80.3%) and only high-quality studies (70.2%) were much lower than the anticipated power of 94.6%(S8L-S8N Fig in S1 File).

For ACS, the subgroup analysis did not reveal any significant covariates. Meta-regression carried out with mean age, % of females, % with diabetes, and % receiving GpIIb/IIIa inhibitors also did not reveal any significant covariates (S4 Table in S1 File) in patients with ACS. For patients with STEMI, % of patients receiving GpIIb/IIIa inhibitors was significantly associated with treatment effect in both subgroup and meta-regression analyses. In trials where ≥25% of patients received GpIIb/IIIa inhibitors, all-cause mortality was 46% lower in the TR group but in trials where <25% received the inhibitors, there was no difference between the TRA and TFA groups (S5 Table and S9 Fig in S1 File).

### Other clinical outcomes

**Fig 4:**
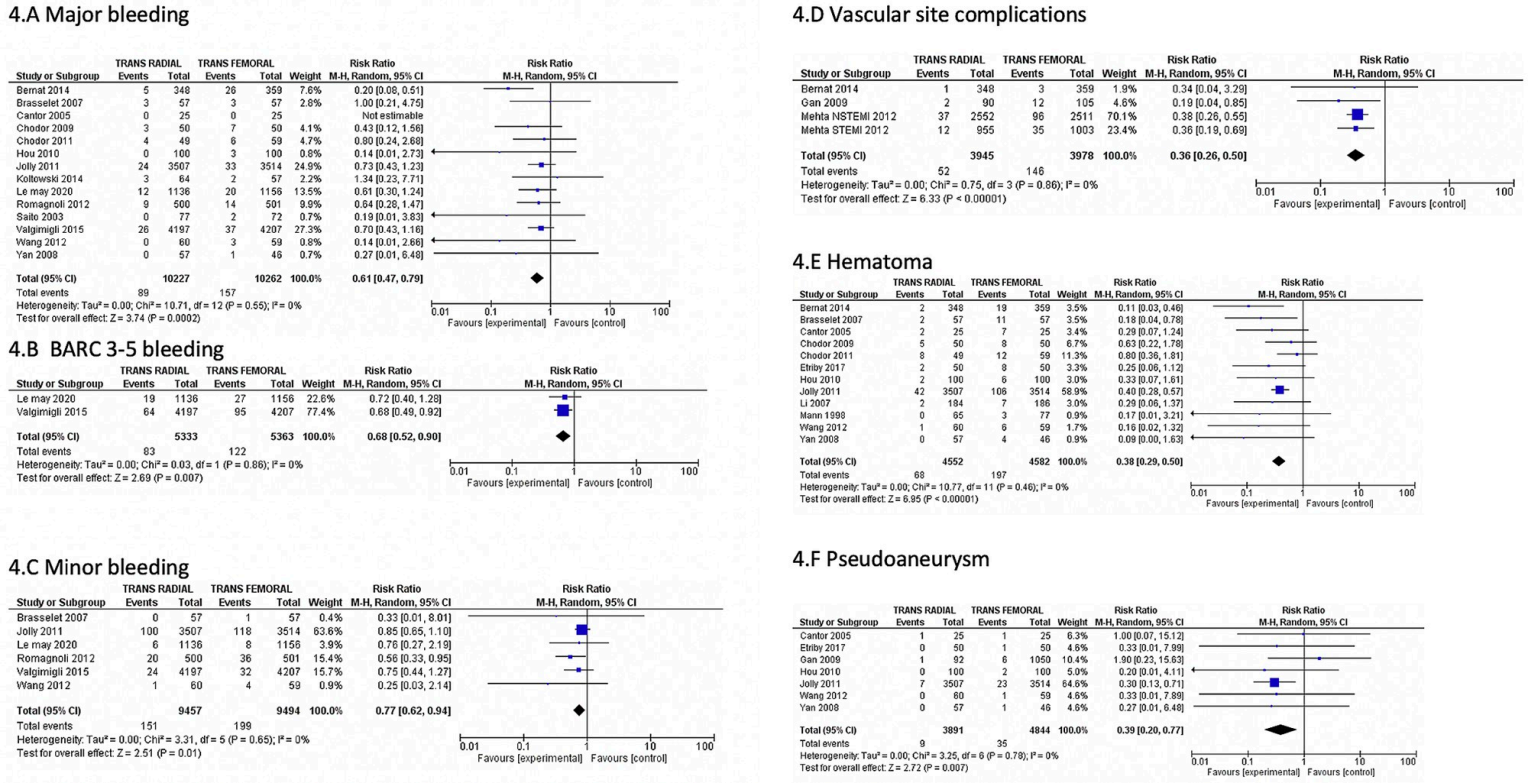
Comparison of Trans-radial approach (TRA) versus Trans-femoral approach (TFA) in patients with acute coronary syndrome showing reduced major bleeding (A), BARC-3-5 bleeding (B), minor bleeding (C), vascular complications (D), hematoma (E) and Pseudoaneurysm (F). BARC- Bleeding academic research consortium; M-H = Mantel-Haenszel; CI- = confidence interval.

**Fig 5:**
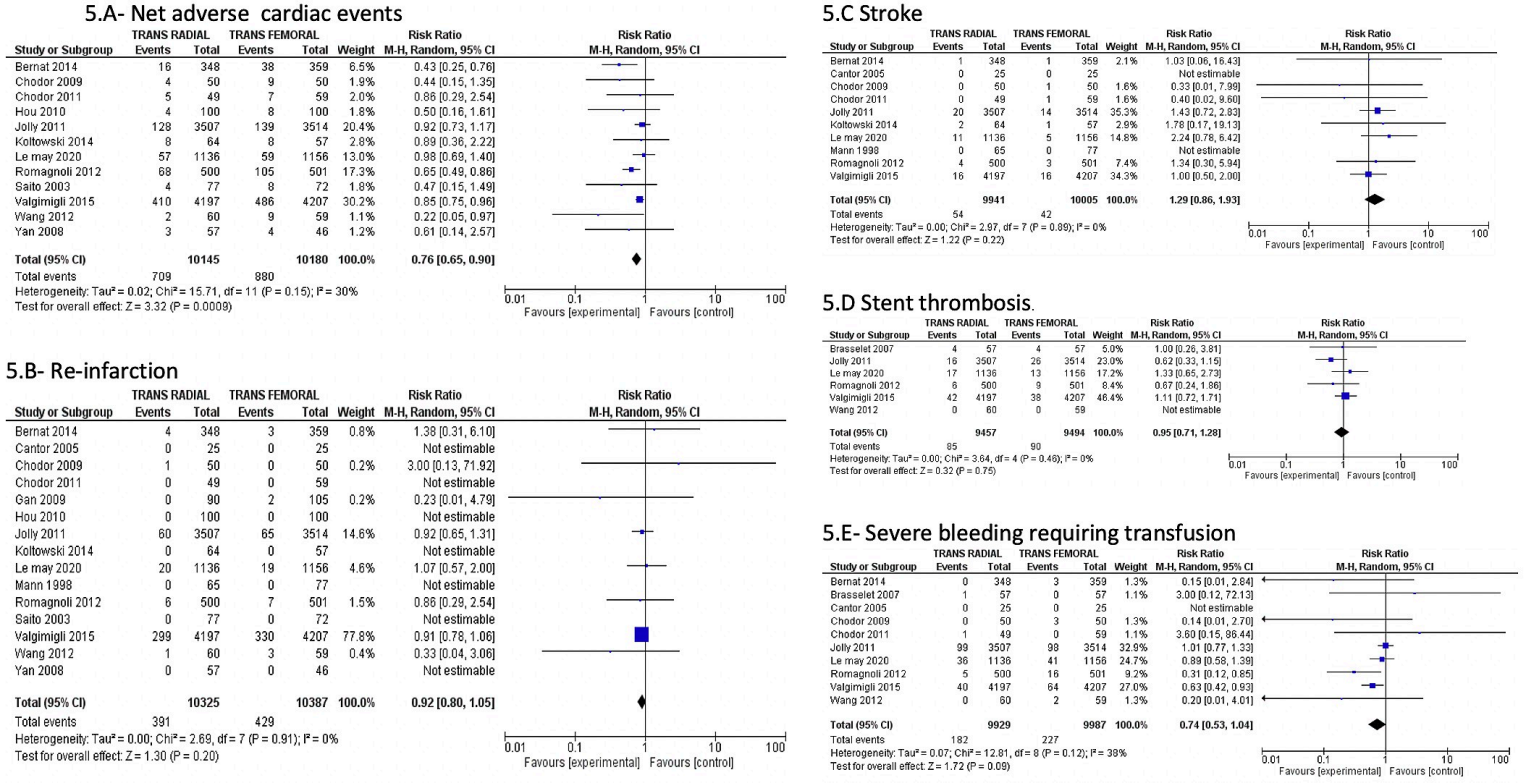
Comparison of Trans-radial approach (TRA) versus Trans-femoral approach (TFA) in patients with acute coronary syndrome showing reduced NACE favoring TRA (A). However, no difference was observed between TRA and TFA on reinfarction (B), stroke (C), stent thrombosis (D) and severe bleeding requiring transfusions (E). NACE- Net adverse cardiac outcomes; M-H = Mantel-Haenszel; CI- = confidence interval.

We found significantly decreased study-defined major bleeding(0.9% versus 1.5%; RR: 0.61; 95% CI: 0.47–0.79; P = 0.0002; I^2^ = 0%), BARC class 3–5 bleeding(1.6% vs 2.3%; RR: 0.68; 95% CI: 0.52–0.90; P = 0.007; I^2^ = 0%), minor bleeding(1.6% versus 2.0%; RR: 0.77; 95% CI: 0.62–0.94; P = 0.01; I^2^ = 0%), vascular site complications(1.3% vs 3.7%; RR: 0.36; 95% CI: 0.26–0.50; P<0.00001; I^2^ = 0%), hematoma(1.5% vs 4.3%; RR: 0.38; 95% CI: 0.29–0.50; P<0.00001; I^2^ = 0%), and pseudoaneurysms(0.2% vs 0.7%; RR: 0.39; 95% CI: 0.20–0.77; P = 0.007; I^2^ = 0%) in the trans-radial arm [Fig 4A–4D & S10 and S11 Figs in S1 File]. We noticed increased NACE in trans-femoral arm mostly due to the effect of study-defined major bleeding [7% versus 8.6%; RR: 0.76; 95% CI: 0.65–0.90; P = 0.0009; I^2^ = 30% Fig 5A]. We did not observe any difference in re-infarction(3.8% versus 4.1%; RR: 0.92; 95% CI: 0.80–1.05; P = 0.20; I^2^ = 0%), stroke(0.5% versus 0.4%; RR: 1.29; 95% CI: 0.86–1.93; P = 0.22; I^2^ = 0%), stent thrombosis(0.9% vs 0.9%; RR: 0.95; 95% CI: 0.71–1.28; P = 0.75; I^2^ = 0%), and severe bleeding requiring blood transfusions between the groups (1.8% vs 2.2%; RR: 0.74; 95% CI: 0.53–1.04; P = 0.09; I^2^ = 38%)[Fig 5B–5D & S12 Fig in S1 File]. Mortality was described as in-hospital mortality in 6 studies and hence, they were analyzed separately. It showed no difference in the in-hospital mortality between the two arms (1.5% versus 2.4%; RR: 0.68; 95% CI: 0.24–1.93; P = 0.47; I^2^ = 0%), S13 Fig in S1 File]. As expected, we observed more access-site crossover with trans-radial procedures [(6.7% vs 2.1%; RR: 3.09; 95% CI: 2.41–3.94; P = <0.00001; I^2^ = 31%]) S14 Fig in S1 File]. We noticed a small decreased contrast volume use (ml) [mean difference (95% CI):−4.6 (−8.5 to −0.7)], small but significantly increased procedural time {mean difference(95% CI) 1.2 [0.1 to 2.3]} and fluoroscopy time {mean difference(95% CI) 0.8 [0.3 to1.4] min} in the trans-radial group (S15-S17 Figs in S1 File). There was no difference in arrival time at PCI to first balloon inflation (FBI) {mean difference(95% CI) 1.9 [−1.3; 5.1] min} (S18 Fig in S1 File). We studied the study defined acute kidney injury and mean difference in creatinine pre and post PCI between the TRA and TFA arms, and found no difference between the groups (S19 & S20 Figs in S1 File).

## Discussion

The main findings of our meta-analysis that included only randomized controlled trials involving patients with acute coronary syndrome are (i) trans-radial procedures were associated with decreased all-cause mortality in patients with ACS undergoing PCI (ii) they are associated with decreased MACE, NACE, study-defined major bleeding, BARC class 3–5 bleeding, vascular complications, hematoma, and pseudoaneurysms without any difference in the in-hospital mortality rate, reinfarction, stroke, MACE, stent thrombosis, and severe bleeding requiring blood transfusions. We also found a significantly reduced 30-day mortality with trans-radial procedures in patients with STEMI alone. Such potential benefits of TRA may be due to reduced bleeding, early ambulation reducing infections and venous thromboembolisms. Our meta-analysis is holistic with separate analysis for the outcomes in patients with ACS and STEMI. We also analyzed the results based on the quality of studies in addition to the calculation for the power of the meta-analysis for clinically important outcomes along with meta-regression of various factors for those outcomes.

None of the included randomized studies were powered for all-cause mortality events. Hence, it is essential to do the metanalysis to know the effect of trans-radial procedures on outcomes. Though a large number of metanalysis is available comparing TRA with TFA, only a few of them are good quality metanalysis conducted in patients with ACS. The metanalysis performed by Ando et al [22] involving only high-quality studies with low risk of bias found reduced mortality, MACE, major bleeding in the trans-radial arm, similar to our results. Though the metanalysis by Ruiz-Rodriguez et al [23] was diluted by the amalgamation of data from RCTs and cohort studies, their result was similar to our results. However, the beneficial effects of trans-radial procedures were questioned by Le May et al who found no difference in 30 days all-cause mortality and MACE between trans-radial and transfemoral arms [4]. It was prematurely stopped, and event rates were lower than expected, resulting in a study underpowered to show any difference between the two arms in terms of mortality. It also needs to be emphasized that the above study used bivalirudin in most of the patients (Table 1), and vascular closure devices were used in more than 2/3^rd^ of the patients in the transfemoral arm. A closer look also showed a significantly reduced use of GpIIb/IIIa inhibitors (only 6%) which is usually low as compared with other studies (Table 1) and real-world practice [24]. We included the above trial in our metanalysis. Despite adding that trial in our meta-analysis, the result did not change. This underscores the beneficial effect of trans-radial PCI in patients with ACS. Ando et al found reduced occurrence of AKI in the TRA as compared with TFA, and found that such a reduced AKI event was predominantly responsible for the reduction in the all-cause mortality [25, 26]. In contrast, in our metanalysis, we did not observe the same. Its needs to be emphasized that the above study was 2x2 factorial one. The sub-group analysis found that such a difference in acute kidney injury was observed in the heparin arm alone without any difference in the bivalirudin arm. This is a hypothesis generating finding as no difference between the arms was observed for the co-primary efficacy and safety end points in the MATRIX- Access or anti-thrombin program [19]. Whether the difference attributed could be because of heparin or bivalirudin needs to further studied in another RCT. Also, when studies were categorized based on ≥25% of patients receiving GpIIb/IIIa inhibitors, no significant difference in adverse clinical outcomes was observed between TRA and TFA groups. This underscores importance of bleeding (access and non-access site) related to them resulting in worse clinical outcomes.

Both MI and bleedings were associated with mortality [27]. Reduction in major bleeding has been shown to have a reduction in ischemic events. Bleeding, not only leads to interruption in anti-platelets, but also causes activation of inflammatory pathways that might lead to increased ischemic events. This is especially important in patients with STEMI where more potent anti-thrombotic would be used. Similar to our metanalysis, Jhand et al [28] have shown that TRA procedures are associated with lower all-cause mortality and bleeding in patients with STEMI. In systems of care where pharmaco-invasive and rescue PCI therapy is utilized for STEMI, TRA acts as a boon to prevent access site-related bleeding complications. Any access site-related bleeding in such a clinical situation that warrants interference in anti-platelet therapy will increase complications. Other possible benefits of TRA include early ambulation that will reduce hospital-related infections and venous-thromboembolism. Though we noticed increased procedural time with TRA, we did not find any difference in the arrival at PCI to the FBI which was in contrast to an analysis of the National Cardiovascular Disease Registry (NCDR) which revealed a modestly increased door-to-balloon time with TRA compared with TFA [29].

Vascular closure devices (VCDs) are increasingly used in interventional cardiology practice. VCDs may decrease the time to ambulation after the procedure. However, several studies including a recent metanalysis have shown that VCDs are not superior to manual compression in safety and efficacy [30, 31]. Also, a recent meta-analysis showed the superiority of the trans-radial procedure over trans-femoral procedures where VCDs were used [32]. Hence, we believe trans-radial procedures should be considered superior to VCD-assisted TFA procedures unless proved otherwise by a sufficiently powered RCT. All the studies included in our meta-analysis have excluded patients with **cardiogenic shock (CS)**. However, Gandhi et al [33] and Pancholy et al [34] showed that the trans-radial procedures reduced 30day mortality and MACE in patients **with CS**. Though it was based on observational studies, it could be extrapolated in patients with ACS **and CS** provided excellent operator experience is available. With the increase in the expertise of the operator and the institution, the ease of doing radial procedures will increase. Adopting a large volume radial procedural program even in patients with STEMI will lead to increasingly available expertise in patients with STEMI and **CS** that may improve patient outcomes.

## Limitations

**First, our study is a study-level meta-analysis of randomized studies and the search strategy was restricted to only articles published in English language and only Pubmed and Embase databases were screened for our meta-analysis. Second, many salient outcomes are not studied by all the available studies (For e.g., B**ARC 3–5 bleeding was reported only in the MATRIX and SAFARI-STEMI trials), and the definition used for some of the outcomes like myocardial infarction and major bleeding differs between the included studies. Third, several of the studies included in the final analysis except five of them as described above had a high-risk of bias or had some-concerns. However, a sub-group analysis restricted to studies with a low risk of bias showed similar results. Fourth, the anti-coagulant used in these trials were not the same (Table 1). However, Valgimigli et al have shown that there was no difference in MACE between bivalirudin and heparin arms [19]. Fifth, variation in the use of GpIIb/IIIa inhibitors, second anti-platelet agent and VCDs were also noticed among the included studies. Sixth, outcomes of radial procedures depend on expertise which was not pre-defined in most of the trials. In spite having many limitations, our metanalysis is the first powered metanalysis that answers the effect of TRA on all-cause mortality. We have described the results separately for patients with ACS and STEMI. In addition, we also analyzed the results based on the quality of studies which is very important to understand the quality of the results. Meta-regression for various factors like mean age, % of females, % with diabetes, and % receiving GpIIb/IIIa inhibitor were also used. Lastly, a properly done ultrasound-guided femoral access with less usage of GpIIb/IIIa inhibitors versus radial access has not been randomly studied. Until then, we may not generalize the results.

## Conclusion

Our metanalysis conclude that in patients with ACS undergoing PCI, trans-radial approach is associated with reduced 30-day all-cause mortality (more so in patients with STEMI), MACE, NACE, study-defined major bleeding, BARC class 3–5 bleeding, vascular complications, hematoma, and pseudo-aneurysms. **Hence, TRA should be considered as a default procedural access strategy in most of the patients with ACS undergoing PCI**. All interventionists should strive hard to master TRA so as to improve patient outcomes.

## Supporting information

S1 File (DOCX)
