# Supplementary material for: Trans-radial approach versus trans-femoral approach in patients with acute coronary syndrome undergoing percutaneous coronary intervention: An updated meta-analysis of randomized controlled trials
Source: PLoS One. 2022 Apr 28;17(4):e0266709. doi: 10.1371/journal.pone.0266709 (PMC9050011; doi:10.1371/journal.pone.0266709)
Supplement: S1 File — (DOCX) [file pone.0266709.s001.docx]

**Supplementary document**

**Key words used for search strategy:**

**Acute coronary syndrome , Percutaneous coronary intervention, PCI, Radial , Femoral**

**Databases searched:**

**Pubmed and Embase**

**Supplementary Table 1.** PRISMA Checklist

| **Section/topic** | **#** | **Checklist item** | **Reported on page #** |
| --- | --- | --- | --- |
| **TITLE** | | |  |
| Title | 1 | Identify the report as a systematic review, meta-analysis, or both. | 1 |
| **ABSTRACT** | | |  |
| Structured summary | 2 | Provide a structured summary including, as applicable: background; objectives; data sources; study eligibility criteria, participants, and interventions; study appraisal and synthesis methods; results; limitations; conclusions and implications of key findings; systematic review registration number. | 2 |
| **INTRODUCTION** | | |  |
| Rationale | 3 | Describe the rationale for the review in the context of what is already known. | 3 |
| Objectives | 4 | Provide an explicit statement of questions being addressed with reference to participants, interventions, comparisons, outcomes, and study design (PICOS). | 3 |
| **METHODS** | | |  |
| Protocol and registration | 5 | Indicate if a review protocol exists, if and where it can be accessed (e.g., Web address), and, if available, provide registration information including registration number. | n/a |
| Eligibility criteria | 6 | Specify study characteristics (e.g., PICOS, length of follow-up) and report characteristics (e.g., years considered, language, publication status) used as criteria for eligibility, giving rationale. | 3-4 |
| Information sources | 7 | Describe all information sources (e.g., databases with dates of coverage, contact with study authors to identify additional studies) in the search and date last searched. | 4 |
| Search | 8 | Present full electronic search strategy for at least one database, including any limits used, such that it could be repeated. | Supplementary page 1 |
| Study selection | 9 | State the process for selecting studies (i.e., screening, eligibility, included in systematic review, and, if applicable, included in the meta-analysis). | 4 |
| Data collection process | 10 | Describe method of data extraction from reports (e.g., piloted forms, independently, in duplicate) and any processes for obtaining and confirming data from investigators. | 4 |
| Data items | 11 | List and define all variables for which data were sought (e.g., PICOS, funding sources) and any assumptions and simplifications made. | 4 and 5 |
| Risk of bias in individual studies | 12 | Describe methods used for assessing risk of bias of individual studies (including specification of whether this was done at the study or outcome level), and how this information is to be used in any data synthesis. | 4 |
| Summary measures | 13 | State the principal summary measures (e.g., risk ratio, difference in means). | 4 and 5 |
| Synthesis of results | 14 | Describe the methods of handling data and combining results of studies, if done, including measures of consistency (e.g., I^2^) for each meta-analysis. | 5 and 6 |

| **Section/topic** | **#** | **Checklist item** | **Reported on page #** |
| --- | --- | --- | --- |
| Risk of bias across studies | 15 | Specify any assessment of risk of bias that may affect the cumulative evidence (e.g., publication bias, selective reporting within studies). | 6 and 7 & Supplementary table.2 |
| Additional analyses | 16 | Describe methods of additional analyses (e.g., sensitivity or subgroup analyses, meta-regression), if done, indicating which were pre-specified. | 5 and 6 |
| **RESULTS** | | |  |
| Study selection | 17 | Give numbers of studies screened, assessed for eligibility, and included in the review, with reasons for exclusions at each stage, ideally with a flow diagram. | Figure 1 |
| Study characteristics | 18 | For each study, present characteristics for whi­­ch data were extracted (e.g., study size, PICOS, follow-up period) and provide the citations. | Table.1 |
| Risk of bias within studies | 19 | Present data on risk of bias of each study and, if available, any outcome level assessment (see item 12). | Supplementary Table.2 |
| Results of individual studies | 20 | For all outcomes considered (benefits or harms), present, for each study: (a) simple summary data for each intervention group (b) effect estimates and confidence intervals, ideally with a forest plot. | Table.1 and Figures.1 to 5 |
| Synthesis of results | 21 | Present results of each meta-analysis done, including confidence intervals and measures of consistency. | Figuress.1 to 5 and Supplementary figures.1-19 |
| Risk of bias across studies | 22 | Present results of any assessment of risk of bias across studies (see Item 15). | Supplementary table.2 |
| Additional analysis | 23 | Give results of additional analyses, if done (e.g., sensitivity or subgroup analyses, meta-regression [see Item 16]). | Supplementary figures.1-19 |
| **DISCUSSION** | | |  |
| Summary of evidence | 24 | Summarize the main findings including the strength of evidence for each main outcome; consider their relevance to key groups (e.g., healthcare providers, users, and policy makers). | 11-13 |
| Limitations | 25 | Discuss limitations at study and outcome level (e.g., risk of bias), and at review-level (e.g., incomplete retrieval of identified research, reporting bias). | 13 |
| Conclusions | 26 | Provide a general interpretation of the results in the context of other evidence, and implications for future research. | 14 |
| **FUNDING** | | |  |
| Funding | 27 | Describe sources of funding for the systematic review and other support (e.g., supply of data); role of funders for the systematic review. | None |

PRISMA = Preferred Reporting Items for Systematic Reviews and Meta-Analyses.

**Supplementary Table.2**

**Table S2.** Risk of Bias Assessment

| **Study details**   \| ***Reference*** \| 1. Bernat I, Horak D, Stasek J, Mates M, Pesek J, Ostadal P, et al. ST-segment elevation myocardial infarction treated by radial or femoral approach in a multicenter randomized clinical trial: the STEMI-RADIAL trial. J Am Coll Cardiol. 2014 Mar 18;63(10):964–72. 2. 2Brasselet C, Tassan S, Nazeyrollas P, Hamon M, Metz D. Randomised comparison of femoral versus radial approach for percutaneous coronary intervention using abciximab in acute myocardial infarction: results of the FARMI trial. Heart. 2007 Dec;93(12):1556–61. 3. Cantor WJ, Puley G, Natarajan MK, Dzavik V, Madan M, Fry A, et al. Radial versus femoral access for emergent percutaneous coronary intervention with adjunct glycoprotein IIb/IIIa inhibition in acute myocardial infarction--the RADIAL-AMI pilot randomized trial. Am Heart J. 2005 Sep;150(3):543–9. 4. Chodór P, Krupa H, Kurek T, Sokal A, Swierad M, Was T, et al. RADIal versus femoral approach for percutaneous coronary interventions in patients with Acute Myocardial Infarction (RADIAMI): A prospective, randomized, single-center clinical trial. Cardiol J. 2009;16(4):332–40. 5. Chodór P, Kurek T, Kowalczuk A, Świerad M, Wąs T, Honisz G, et al. Radial vs femoral approach with StarClose clip placement for primary percutaneous coronary intervention in patients with ST-elevation myocardial infarction. RADIAMI II: a prospective, randomised, single centre trial. Kardiol Pol. 2011;69(8):763–71. 6. Etriby SE, Nassar A, Rifaie O, Mahmoudy AE, Missiry AE. The impact of transradial vs transfemoral approach for percutaneous coronary intervention on the outcome of patients presenting with acute coronary syndrome. J Invasive Cardiol [Internet]. 2017;29(10):E119. Available from: <https://www.embase.com/search/results?subaction=viewrecord&id=L620355682&from=export> 7. Gan L, Lib Q, Liuc R, Zhaoc Y, Qiuc J, Liao Y. Effectiveness and feasibility of transradial approaches for primary percutaneous coronary intervention in patients with acute myocardial infarction. Journal of Nanjing Medical University [Internet]. 2009 Jul 1 [cited 2021 Jan 18];23(4):270–4. Available from: <http://www.sciencedirect.com/science/article/pii/S100743760960068X> 8. Hou L, Wei Y-D, Li W-M, Xu Y-W. Comparative study on transradial versus transfemoral approach for primary percutaneous coronary intervention in Chinese patients with acute myocardial infarction. Saudi Med J. 2010 Feb;31(2):158–62. 9. Jolly SS, Yusuf S, Cairns J, Niemelä K, Xavier D, Widimsky P, et al. Radial versus femoral access for coronary angiography and intervention in patients with acute coronary syndromes (RIVAL): A randomised, parallel group, multicentre trial. Lancet [Internet]. 2011;377(9775):1409–20. Available from: <https://www.embase.com/search/results?subaction=viewrecord&id=L51348599&from=export> 10. Kołtowski L, Filipiak KJ, Kochman J, Pietrasik A, Rdzanek A, Huczek Z, et al. Access for percutaneous coronary intervention in ST segment elevation myocardial infarction: radial vs. femoral--a prospective, randomised clinical trial (OCEAN RACE). Kardiol Pol. 2014;72(7):604–11. 11. Le May M, Wells G, So D, Chong AY, Dick A, Froeschl M, et al. Safety and Efficacy of Femoral Access vs Radial Access in ST-Segment Elevation Myocardial Infarction: The SAFARI-STEMI Randomized Clinical Trial. JAMA Cardiol. 2020 Feb 1;5(2):126–34. 12. Li W, Li Y, Zhao J, Duan Y, Sheng L, Yang B, et al. Safety and feasibility of emergent percutaneous coronary intervention with the transradial access in patients with acute myocardial infarction. Chin Med J (Engl). 2007 Apr 5;120(7):598–600. 13. Mann T, Cubeddu G, Bowen J, Schneider JE, Arrowood M, Newman WN, et al. Stenting in acute coronary syndromes: a comparison of radial versus femoral access sites. J Am Coll Cardiol. 1998 Sep;32(3):572–6. 14. Romagnoli E, Biondi-Zoccai G, Sciahbasi A, Politi L, Rigattieri S, Pendenza G, et al. Radial versus femoral randomized investigation in st-segment elevation acute coronary syndrome: The rifle-steacs (radial versus femoral randomized investigation in st-elevation acute coronary syndrome) study. J Am Coll Cardiol [Internet]. 2012;60(24):2481–9. Available from: <https://www.embase.com/search/results?subaction=viewrecord&id=L52140946&from=export> 15. Saito S, Tanaka S, Hiroe Y, Miyashita Y, Takahashi S, Tanaka K, et al. Comparative study on transradial approach vs. transfemoral approach in primary stent implantation for patients with acute myocardial infarction: results of the test for myocardial infarction by prospective unicenter randomization for access sites (TEMPURA) trial. Catheter Cardiovasc Interv. 2003 May;59(1):26–33. 16. Valgimigli M, Frigoli E, Leonardi S, Rothenbühler M, Gagnor A, Calabrò P, et al. Bivalirudin or Unfractionated Heparin in Acute Coronary Syndromes. N Engl J Med. 2015 Sep 10;373(11):997–1009. 17. Wang Y-B, Fu X-H, Wang X-C, Gu X-S, Zhao Y-J, Hao G-Z, et al. Randomized comparison of radial versus femoral approach for patients with STEMI undergoing early PCI following intravenous thrombolysis. J Invasive Cardiol. 2012 Aug;24(8):412–6. 18. Yan Z-X, Zhou Y-J, Zhao Y-X, Liu Y-Y, Shi D-M, Guo Y-H, et al. Safety and feasibility of transradial approach for primary percutaneous coronary intervention in elderly patients with acute myocardial infarction. Chin Med J [Internet]. 2008;121(9):782–6. Available from: https://www.embase.com/search/results?subaction=viewrecord&id=L352284870&from=export \| \| --- \| --- \|   **Study design**   \| ⮽ \| Individually-randomized parallel-group trial \| \| --- \| --- \| \| □ \| Cluster-randomized parallel-group trial \| \| □ \| Individually randomized cross-over (or other matched) trial \|  \| **Specify which outcome is being assessed for risk of bias** \| All-cause mortality at 30 days \| \| --- \| --- \|  \| **Specify the numerical result being assessed**. \| RE model; RR, 0.81 ;95% CI [0.69-0.95 ]  P=0.01; I^2^=0% (Central Illustration) \| \| --- \| --- \|   **Is the review team’s aim for this result…?**   \| ⮽ \| to assess the effect of *assignment to intervention* (the ‘intention-to-treat’ effect) \| \| --- \| --- \| \| □ \| to assess the effect of *adhering to intervention* (the ‘per-protocol’ effect) \|   **Which of the following sources were obtained to help inform the risk-of-bias assessment? (tick as many as apply)**  ⮽ Journal article(s) with results of the trial  ⮽ Trial protocol  ⮽ Statistical analysis plan (SAP)  □ Non-commercial trial registry record (e.g. ClinicalTrials.gov record)  □ Company-owned trial registry record (e.g. GSK Clinical Study Register record)  □ “Grey literature” (e.g. unpublished thesis)  □ Conference abstract(s) about the trial  □ Regulatory document (e.g. Clinical Study Report, Drug Approval Package)  □ Research ethics application  □ Grant database summary (e.g. NIH RePORTER or Research Councils UK Gateway to Research)  □ Personal communication with trialist  □ Personal communication with the sponsor |
| --- | --- | --- | --- | --- | --- | --- | --- | --- | --- | --- | --- | --- | --- | --- | --- | --- |

**Part-1**

**Domain 1: Risk of bias arising from the randomization process**

| **Signaling questions** | Bernet et al | Brasslet et al | Cantor et al | Chodor et al 2009 | Chodor et al 2011 | Etriby et al | Gan et al |
| --- | --- | --- | --- | --- | --- | --- | --- |
| **1.1 Was the allocation sequence random?** | Y | Y | Y | Y | Y | Y | Y |
| **1.2 Was the allocation sequence concealed until participants were enrolled and assigned to interventions?** | Y | NI | NI | NI | NI | NI | NI |
| **1.3 Did baseline differences between intervention groups suggest a problem with the randomization process?** | N | N | N | **N** | N | N | N |
| **Risk-of-bias judgement** | Low | SOME CONCERNS | SOME CONCERNS | SOME CONCERNS | SOME CONCERNS | SOME CONCERNS | SOME CONCERNS |
| Optional: What is the predicted direction of bias arising from the randomization process? | - | - | - | - | - | - | - |

Domain 2: Risk of bias due to deviations from the intended interventions (*effect of assignment to intervention*)

| **Signaling questions** | Bernet et al | Brasslet et al | Cantor et al | Chodor et al 2009 | Chodor et al 2011 | Etriby et al | Gan et al |
| --- | --- | --- | --- | --- | --- | --- | --- |
| **2.1. Were participants aware of their assigned intervention during the trial?** | Y | Y | Y | Y | Y | Y | Y |
| **2.2. Were carers and people delivering the interventions aware of participants' assigned intervention during the trial?** | Y | Y | Y | Y | Y | Y | Y |
| **2.3. If Y/PY/NI to 2.1 or 2.2: Were there deviations from the intended intervention that arose because of the experimental context?** | N | N | N | N | N | N | N |
| **2.4. If Y/PY to 2.3: Were these deviations from intended intervention balanced between groups?** | - | - | - | - | - | - | - |
| **2.5 If N/PN/NI to 2.4: Were these deviations likely to have affected the outcome?** | - | - | - | - | - | - | - |
| **2.6 Was an appropriate analysis used to estimate the effect of assignment to intervention?** | Y | Y | Y | Y | Y | Y | Y |
| **2.7 If N/PN/NI to 2.6: Was there potential for a substantial impact (on the result) of the failure to analyze participants in the group to which they were randomized?** | - | - | - | - | - | - | - |
| **Risk-of-bias judgement** | LOW | LOW | LOW | LOW | LOW | LOW | LOW |
| Optional: What is the predicted direction of bias due to deviations from intended interventions? |  |  |  |  |  |  |  |
|  |  |  |  |  |  |  |  |

Domain 2: Risk of bias due to deviations from the intended interventions (*effect of adhering to intervention*)

| **Signaling questions** | Bernet et al | Brasslet et al | Cantor et al | Chodor et al 2009 | Chodor et al 2011 | Etriby et al | Gan et al |
| --- | --- | --- | --- | --- | --- | --- | --- |
| **2.1. Were participants aware of their assigned intervention during the trial?** | Y | Y | Y | Y | Y | Y | Y |
| **2.2. Were carers and people delivering the interventions aware of participants' assigned intervention during the trial?** | Y | Y | Y | Y | Y | Y | Y |
| **2.3. If Y/PY/NI to 2.1 or 2.2: Were important co-interventions balanced across intervention groups?** | Y | Y | Y | Y | Y | Y | Y |
| **2.4. Could failures in implementing the intervention have affected the outcome?** | N | N | N | N | N | N | N |
| **2.5. Did study participants adhere to the assigned intervention regimen?** | Y | Y | Y | Y | Y | Y | Y |
| **2.6. If N/PN/NI to 2.3 or 2.5 or Y/PY/NI to 2.4: Was an appropriate analysis used to estimate the effect of adhering to the intervention?** | Y | Y | Y | Y | Y | Y | Y |
| **Risk-of-bias judgement** | LOW | LOW | LOW | LOW | LOW | LOW | LOW |

Domain 3: Missing outcome data

| **Signaling questions** | Bernet et al | Brasslet et al | Cantor et al | Chodor et al 2009 | Chodor et al 2011 | Etriby et al | Gan et al |
| --- | --- | --- | --- | --- | --- | --- | --- |
| **3.1 Were data for this outcome available for all, or nearly all, participants randomized?** | Y | Y | N | Y | Y | Y | N |
| **3.2 If N/PN/NI to 3.1: Is there evidence that result was not biased by missing outcome data?** | - | - | N | -- | - | -- | PN |
| **3.3 If N/PN to 3.2: Could missingness in the outcome depend on its true value?** | - | - | PY | - | - | - | NI |
| **3.4 If Y/PY/NI to 3.3: Do the proportions of missing outcome data differ between intervention groups?** | - | - | PY | - | - | - | NI |
| **3.5 If Y/PY/NI to 3.3: Is it likely that missingness in the outcome depended on its true value?** | - | - | PY | - | - | - | NI |
| **Risk-of-bias judgement** | Low | Low | HIGH | Low | Low | Low | SOME CONCERNS |

Domain 4: Risk of bias in measurement of the outcome

| **Signaling questions** | Bernet et al | Brasslet et al | Cantor et al | Chodor et al 2009 | Chodor et al 2011 | Etriby et al | Gan et al |
| --- | --- | --- | --- | --- | --- | --- | --- |
| **4.1 Was the method of measuring the outcome inappropriate?** | N | N | N | N | N | N | N |
| **4.2 Could measurement or ascertainment of the outcome have differed between intervention groups ?** | N | N | N | N | N | N | N |
| **4.3 If N/PN/NI to 4.1 and 4.2: Were outcome assessors aware of the intervention received by study participants ?** | - | - | - | - | - | - | - |
| **4.4 If Y/PY/NI to 4.3: Could assessment of the outcome have been influenced by knowledge of intervention received?** | - | - | - | - | - | - | - |
| **4.5 If Y/PY/NI to 4.4: Is it likely that assessment of the outcome was influenced by knowledge of intervention received?** | - | - | - | - | - | - | - |
| **Risk-of-bias judgement** | Low | Low | Low | Low | Low | Low | Low |

Domain 5: Risk of bias in selection of the reported result

| **Signaling questions** | Bernet et al | Brasslet et al | Cantor et al | Chodor et al 2009 | Chodor et al 2011 | Etriby et al | Gan et al |
| --- | --- | --- | --- | --- | --- | --- | --- |
| **5.1 Was the trial analyzed in accordance with a pre-specified plan that was finalized before unblinded outcome data were available for analysis ?** | Y | N | Y | Y | Y | Y | Y |
| **5.2 Is the numerical result being assessed likely to have been selected, on the basis of the results, from ….multiple outcome measurements (e.g. scales, definitions, time points) within the outcome domain?** | N | Y | N | N | N | N | N |
| **5.3 ... multiple analyses of the data?** | N | Y | N | N | N | Y | N |
| **Risk-of-bias judgement** | Low | HIGH | Low | LOW | LOW | LOW | Low |
| Optional: What is the predicted direction of bias due to selection of the reported result? |  |  |  |  |  |  |  |

Overall risk of bias

|  | Bernet et al | Brasslet et al | Cantor et al | Chodor et al 2009 | Chodor et al 2011 | Etriby et al | Gan et al |
| --- | --- | --- | --- | --- | --- | --- | --- |
| **Risk of Bias judgement** | LOW | HIGH | HIGH | Some concern | Some concern | Some concern | Some concern |

| **Signaling questions** | Hou et al | Jolly et al | Koltowski et al | Le may et al | Li et al | Mann et al |
| --- | --- | --- | --- | --- | --- | --- |
| **1.1 Was the allocation sequence random?** | Y | Y | Y | Y | Y | Y |
| **1.2 Was the allocation sequence concealed until participants were enrolled and assigned to interventions?** | NI | Y | NI | Y | NI | NI |
| **1.3 Did baseline differences between intervention groups suggest a problem with the randomization process?** | N | N | N | N | N | N |
| **Risk-of-bias judgement** | SOME CONCERNS | LOW | SOME CONCERNS | Low | SOME CONCERNS | SOME CONCERNS |
| Optional: What is the predicted direction of bias arising from the randomization process? | - | - | - | - | - | - |

**Domain 1: Risk of bias arising from the randomization process**

Domain 2: Risk of bias due to deviations from the intended interventions (*effect of assignment to intervention*)

| **Signaling questions** | Hou et al | Jolly et al | Koltowski et al | Le may et al | Li et al | Mann et al |
| --- | --- | --- | --- | --- | --- | --- |
| **2.1. Were participants aware of their assigned intervention during the trial?** | Y | Y | Y | Y | Y | Y |
| **2.2. Were carers and people delivering the interventions aware of participants' assigned intervention during the trial?** | Y | Y | Y | Y | Y | Y |
| **2.3. If Y/PY/NI to 2.1 or 2.2: Were there deviations from the intended intervention that arose because of the experimental context?** | N | N | PY | N | N | N |
| **2.4. If Y/PY to 2.3: Were these deviations from intended intervention balanced between groups?** | - | - | NI | - | - | - |
| **2.5 If N/PN/NI to 2.4: Were these deviations likely to have affected the outcome?** | - | - | PN | - | - | - |
| **2.6 Was an appropriate analysis used to estimate the effect of assignment to intervention?** | Y | Y | Y | Y | Y | Y |
| **2.7 If N/PN/NI to 2.6: Was there potential for a substantial impact (on the result) of the failure to analyze participants in the group to which they were randomized?** | - | - | - |  | - | - |
| **Risk-of-bias judgement** | LOW | LOW | Some concerns | LOW | LOW | LOW |
| Optional: What is the predicted direction of bias due to deviations from intended interventions? |  |  |  |  |  |  |
|  |  |  |  |  |  |  |

Domain 2: Risk of bias due to deviations from the intended interventions (*effect of adhering to intervention*)

| **Signaling questions** | Hou et al | Jolly et al | Koltowski et al | Le may et al | Li et al | Mann et al |
| --- | --- | --- | --- | --- | --- | --- |
| **2.1. Were participants aware of their assigned intervention during the trial?** | Y | Y | Y | Y | Y | Y |
| **2.2. Were carers and people delivering the interventions aware of participants' assigned intervention during the trial?** | Y | Y | Y | Y | Y | Y |
| **2.3. If Y/PY/NI to 2.1 or 2.2: Were important co-interventions balanced across intervention groups?** | Y | Y | Y | Y | Y | Y |
| **2.4. Could failures in implementing the intervention have affected the outcome?** | N | N | N | N | N | N |
| **2.5. Did study participants adhere to the assigned intervention regimen?** | Y | Y | Y | Y | Y | Y |
| **2.6. If N/PN/NI to 2.3 or 2.5 or Y/PY/NI to 2.4: Was an appropriate analysis used to estimate the effect of adhering to the intervention?** | Y | Y | Y | Y | Y | Y |
| **Risk-of-bias judgement** | LOW | LOW | LOW | LOW | LOW | LOW |

,

Domain 3: Missing outcome data

| **Signaling questions** | Hou et al | Jolly et al | Koltowski et al | Le may et al | Li et al | Mann et al |
| --- | --- | --- | --- | --- | --- | --- |
| **3.1 Were data for this outcome available for all, or nearly all, participants randomized?** | Y | Y | Y | Y | Y | N |
| **3.2 If N/PN/NI to 3.1: Is there evidence that result was not biased by missing outcome data?** | - | - |  |  |  | PN |
| **3.3 If N/PN to 3.2: Could missingness in the outcome depend on its true value?** | - | - |  |  |  | NI |
| **3.4 If Y/PY/NI to 3.3: Do the proportions of missing outcome data differ between intervention groups?** | - | - |  |  |  | NI |
| **3.5 If Y/PY/NI to 3.3: Is it likely that missingness in the outcome depended on its true value?** | - | - |  |  |  | PY |
| **Risk-of-bias judgement** | Low | Low | Low | Low | Low | HIGH |

Domain 4: Risk of bias in measurement of the outcome

| **Signaling questions** | Hou et al | Jolly et al | Koltowski et al | Le may et al | Li et al | Mann et al |
| --- | --- | --- | --- | --- | --- | --- |
| **4.1 Was the method of measuring the outcome inappropriate?** | N | N | N | N | N | N |
| **4.2 Could measurement or ascertainment of the outcome have differed between intervention groups ?** | N | N | N | N | N | N |
| **4.3 If N/PN/NI to 4.1 and 4.2: Were outcome assessors aware of the intervention received by study participants ?** | - | - |  |  |  |  |
| **4.4 If Y/PY/NI to 4.3: Could assessment of the outcome have been influenced by knowledge of intervention received?** | - | - |  |  |  |  |
| **4.5 If Y/PY/NI to 4.4: Is it likely that assessment of the outcome was influenced by knowledge of intervention received?** | - | - |  |  |  |  |
| **Risk-of-bias judgement** | Low | Low | Low | Low | Low | Low |

Domain 5: Risk of bias in selection of the reported result

| **Signaling questions** | Hou et al | Jolly et al | Koltowski et al | Le may et al | Li et al | Mann et al |
| --- | --- | --- | --- | --- | --- | --- |
| **5.1 Was the trial analyzed in accordance with a pre-specified plan that was finalized before unblinded outcome data were available for analysis ?** | Y | Y | Y | Y | N | Y |
| **Is the numerical result being assessed likely to have been selected, on the basis of the results, from...** |  |  |  |  |  |  |
| **5.2. ... multiple outcome measurements (e.g. scales, definitions, time points) within the outcome domain?** | N | N | N | N | NI | N |
| **5.3 ... multiple analyses of the data?** | N | N | N | N | NI | N |
| **Risk-of-bias judgement** | Low | Low | Low | LOW | HIGH | LOW |
| Optional: What is the predicted direction of bias due to selection of the reported result? |  |  |  |  |  |  |

|  | Hou et al | Jolly et al | Koltowski et al | Le may et al | Li et al | Mann et al |
| --- | --- | --- | --- | --- | --- | --- |
| **Risk of Bias judgement** | SOME CONCERNS | LOW RISK | SOME CONCERN | LOW RISK | HIGH RISK | HIGH RISK |

Overall risk of bias

**Domain 1: Risk of bias arising from the randomization process**

| **Signaling questions** | Romagnoli et al | Saito et al | Valgimigli | Wang | Yan |
| --- | --- | --- | --- | --- | --- |
| **1.1 Was the allocation sequence random?** | Y | Y | Y | Y | Y |
| **1.2 Was the allocation sequence concealed until participants were enrolled and assigned to interventions?** | Y | NI | Y | Y | NI |
| **1.3 Did baseline differences between intervention groups suggest a problem with the randomization process?** | N | N | N | N | N |
| **Risk-of-bias judgement** | Low | SOME CONCERNS | Low | Low | SOME CONCERNS |
| Optional: What is the predicted direction of bias arising from the randomization process? | - | - | - | - | - |

Domain 2: Risk of bias due to deviations from the intended interventions (*effect of assignment to intervention*)

| **Signaling questions** | Romagnoli et al | Saito et al | Valgimigli | Wang | Yan |
| --- | --- | --- | --- | --- | --- |
| **2.1. Were participants aware of their assigned intervention during the trial?** | Y | Y | Y | Y | Y |
| **2.2. Were carers and people delivering the interventions aware of participants' assigned intervention during the trial?** | Y | Y | Y | Y | Y |
| **2.3. If Y/PY/NI to 2.1 or 2.2: Were there deviations from the intended intervention that arose because of the experimental context?** | N | Y | Y | Y | Y |
| **2.4. If Y/PY to 2.3: Were these deviations from intended intervention balanced between groups?** | - | NI | PN | NI | NI |
| **2.5 If N/PN/NI to 2.4: Were these deviations likely to have affected the outcome?** | - | NI | PN | NI | NI |
| **2.6 Was an appropriate analysis used to estimate the effect of assignment to intervention?** | Y | Y | Y | Y | Y |
| **2.7 If N/PN/NI to 2.6: Was there potential for a substantial impact (on the result) of the failure to analyze participants in the group to which they were randomized?** | - | - | - | - | - |
| **Risk-of-bias judgement** | LOW | Some concerns | HIGH | Some concerns | Some concerns |
| Optional: What is the predicted direction of bias due to deviations from intended interventions? |  |  |  |  |  |
|  |  |  |  |  |  |

Domain 2: Risk of bias due to deviations from the intended interventions (*effect of adhering to intervention*)

| **Signaling questions** | Romagnoli et al | Saito et al | Valgimigli | Wang | Yan |
| --- | --- | --- | --- | --- | --- |
| **2.1. Were participants aware of their assigned intervention during the trial?** | Y | Y | Y | Y | Y |
| **2.2. Were carers and people delivering the interventions aware of participants' assigned intervention during the trial?** | Y | Y | Y | Y | Y |
| **2.3. If Y/PY/NI to 2.1 or 2.2: Were important co-interventions balanced across intervention groups?** | Y | Y | Y | Y | Y |
| **2.4. Could failures in implementing the intervention have affected the outcome?** | N | N | N | N | N |
| **2.5. Did study participants adhere to the assigned intervention regimen?** | Y | Y | Y | Y | Y |
| **2.6. If N/PN/NI to 2.3 or 2.5 or Y/PY/NI to 2.4: Was an appropriate analysis used to estimate the effect of adhering to the intervention?** | Y | Y | Y | Y | Y |
| **Risk-of-bias judgement** | Some concerns | Some concerns | Some concerns | Some concerns | Some concerns |

Domain 3: Missing outcome data

| **Signaling questions** | Romagnoli et al | Saito et al | Valgimigli | Wang | Yan |
| --- | --- | --- | --- | --- | --- |
| **3.1 Were data for this outcome available for all, or nearly all, participants randomized?** | Y | Y | Y | Y | Y |
| **3.2 If N/PN/NI to 3.1: Is there evidence that result was not biased by missing outcome data?** |  |  |  |  |  |
| **3.3 If N/PN to 3.2: Could missingness in the outcome depend on its true value?** |  |  |  |  |  |
| **3.4 If Y/PY/NI to 3.3: Do the proportions of missing outcome data differ between intervention groups?** |  |  |  |  |  |
| **3.5 If Y/PY/NI to 3.3: Is it likely that missingness in the outcome depended on its true value?** |  |  |  |  |  |
| **Risk-of-bias judgement** | Low | Low | Low | Low | Low |

Domain 4: Risk of bias in measurement of the outcome

| **Signaling questions** | Romagnoli et al | Saito et al | Valgimigli | Wang | Yan |
| --- | --- | --- | --- | --- | --- |
| **4.1 Was the method of measuring the outcome inappropriate?** | N | N | N | N | N |
| **4.2 Could measurement or ascertainment of the outcome have differed between intervention groups ?** | N | N | N | N | N |
| **4.3 If N/PN/NI to 4.1 and 4.2: Were outcome assessors aware of the intervention received by study participants ?** |  |  |  |  |  |
| **4.4 If Y/PY/NI to 4.3: Could assessment of the outcome have been influenced by knowledge of intervention received?** |  |  |  |  |  |
| **4.5 If Y/PY/NI to 4.4: Is it likely that assessment of the outcome was influenced by knowledge of intervention received?** |  |  |  |  |  |
| **Risk-of-bias judgement** | Low | Low | Low | Low | Low |

Domain 5: Risk of bias in selection of the reported result

| **Signaling questions** | Romagnoli et al | Saito et al | Valgimigli | Wang | Yan |
| --- | --- | --- | --- | --- | --- |
| **5.1 Was the trial analyzed in accordance with a pre-specified plan that was finalized before unblinded outcome data were available for analysis ?** | Y | Y | Y | Y | Y |
| **Is the numerical result being assessed likely to have been selected, on the basis of the results, from...** |  |  |  |  |  |
| **5.2. ... multiple outcome measurements (e.g. scales, definitions, time points) within the outcome domain?** | N | N | N | N | N |
| **5.3 ... multiple analyses of the data?** | N | N | N | N | N |
| **Risk-of-bias judgement** | Low | Low | Low | Low | Low |
| Optional: What is the predicted direction of bias due to selection of the reported result? |  |  |  |  |  |

Overall risk of bias

|  | Romagnoli et al | Saito et al | Valgimigli | Wang | Yan |
| --- | --- | --- | --- | --- | --- |
| **Risk of Bias judgement** | LOW RISK | SOME CONCERNS | LOW RISK | SOME CONCERN | Some concern |

**
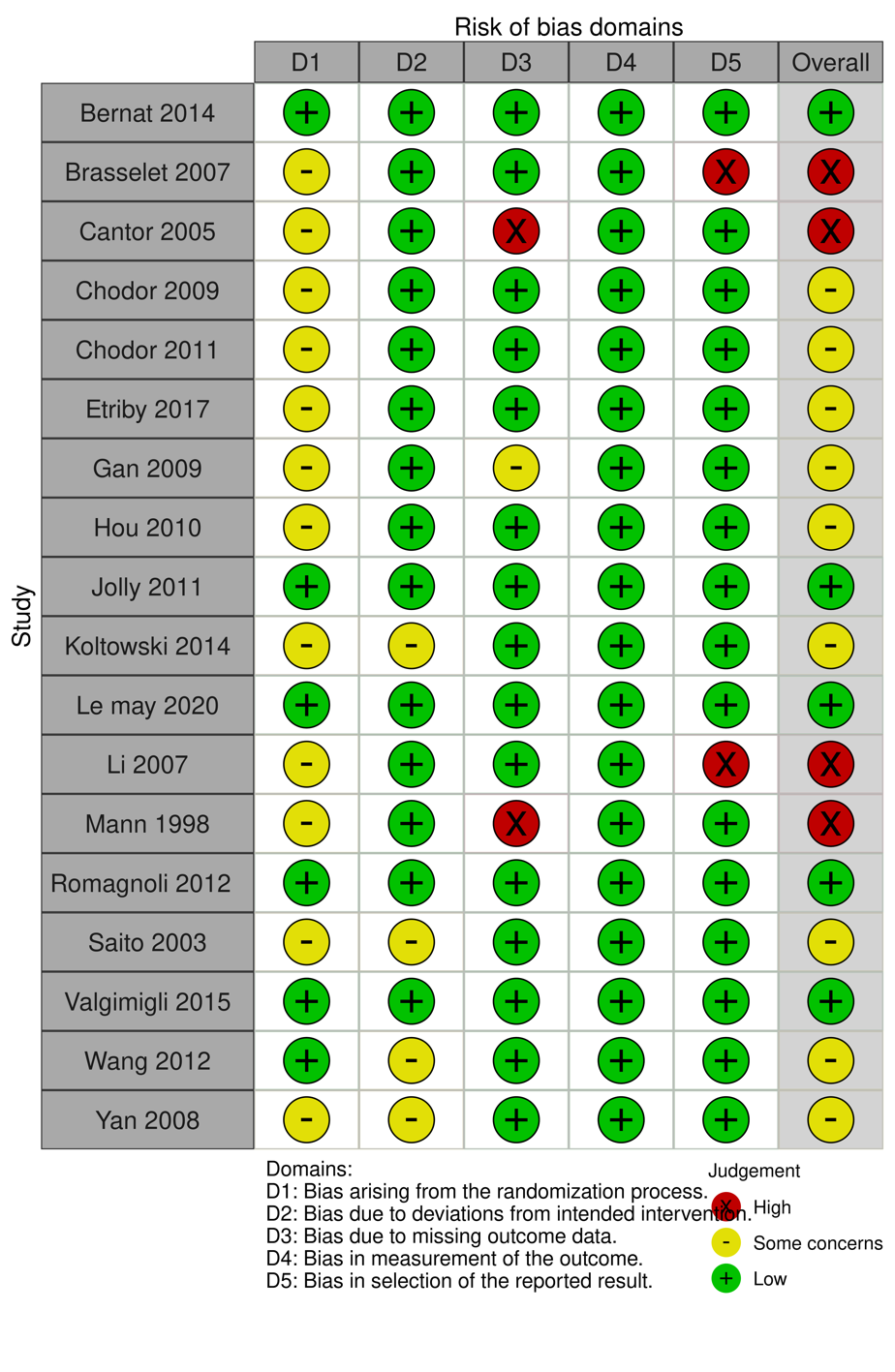
**

**Summary of Supplementary Table.2 Overall Risk of Bias**

**
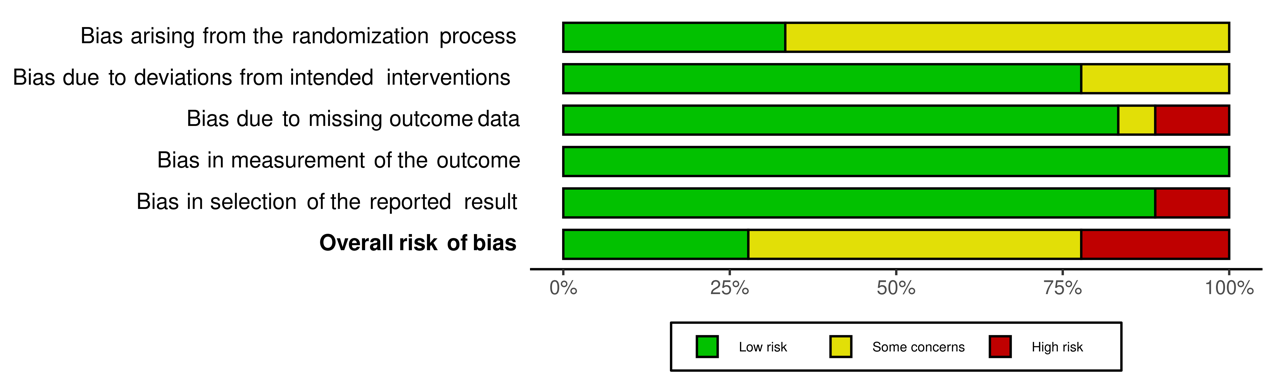
**

**Supplementary Table.3. Sensitivity Analysis Using Fixed-Effect Models**

| **Outcome** | **RR (95% CI)** | **P Value** | **I^2^** |
| --- | --- | --- | --- |
| All-Cause Mortality 30 days | 0.75 [0.62, 0.91] | 0.004 | 0 |
| MACE | 0.87 [0.78, 0.96] | 0.009 | 0 |
| NACE | 0.81 [0.74, 0.89] | <0.00001 | 30 |
| Re infarction | 0.92 [0.80, 1.04] | 0.19 | 0 |
| Stroke | 1.29 [0.86, 1.92] | 0.22 | 0 |
| Stent thrombosis | 0.95 [0.71, 1.27] | 0.73 | 0 |
| Major Bleeding | 0.58 [0.45, 0.75] | <0.0001 | 0 |
| Minor bleeding | 0.76 [0.62, 0.94] | 0.01 | 0 |
| Barc 3-5 | 0.68 [0.52, 0.90] | 0.007 | 0 |
| Haematoma | 0.35 [0.27, 0.46] | <0.00001 | 0 |
| CABG/ Revascularisation | 1.09 [0.96, 1.24] | 0.19 | 0 |
| Intracranial bleeding | 1.68 [0.22, 12.69] | 0.61 | 0 |
| Access cross over | 3.13 [2.69, 3.64] | <0.00001 | 0 |
| Non infarct related PCI | 0.91 [0.71, 1.18] | 0.48 | 0 |
| Severe bleeding requiring transfusion | 0.81 [0.67, 0.98] | 0.03 | 38 |
| Vascular site complications | 0.36 [0.26, 0.49] | <0.00001 | 0 |
| Pseudo aneurysm | 0.37 [0.19, 0.71] | 0.003 | 0 |

|  | **n** | **Bivariate analysis** | | **Meta-regression** | |
| --- | --- | --- | --- | --- | --- |
|  |  | **RR**  **(95% CI)** | **P value** | **Adjusted RR**  **(95% CI)** | **P value** |
| **Age group** |  |  |  |  |  |
| <65 years | 5 | 0.89 (0.65, 1.22) |  | Reference |  |
| ≥65 years | 5 | 0.68 (0.53, 0.87) | 0.181 | 0.84 (0.51, 1.40) | 0.505 |
| **% of females** |  |  |  |  |  |
| <25 | 6 | 0.87 (0.56, 1.38) |  | Reference |  |
| ≥25 | 4 | 0.73 (0.59, 0.90) | 0.470 | 0.9 (0.36, 2.27) | 0.830 |
| **% of patients with diabetes** |  |  |  |  |  |
| <20 | 2 | 1.06 (0.58, 1.95) |  | Reference |  |
| ≥20 | 8 | 0.72 (0.59, 0.89) | 0.238 | 0.85 (0.34, 2.10) | 0.719 |
| **% of patient with hypertension** |  |  |  |  |  |
| <50 | 5 | 0.97 (0.57, 1.65) |  |  |  |
| ≥50 | 5 | 0.72 (0.59, 0.89) | 0.315 | Not included |  |
| **% of smokers** |  |  |  |  |  |
| <50 | 6 | 0.77 (0.60, 1.00) |  |  |  |
| ≥50 | 4 | 0.73 (0.54, 0.97) | 0.755 | Not included |  |
| **% receiving GP 2b/3a inhibitors** |  |  |  |  |  |
| <25 | 4 | 0.81 (0.65, 1.02) |  | Reference |  |
| ≥25 | 4 | 0.6 (0.40, 0.90) | 0.205 | 0.77 (0.45, 1.33) | 0.353 |

**Supplementary Table 4. Subgroup and meta-regression analysis for all-cause mortality among ACS**

**Supplementary Table 5. Subgroup and meta-regression analysis for all-cause mortality among STEMI**

|  | **n** | **Bivariate analysis** | | **Meta-regression** | |
| --- | --- | --- | --- | --- | --- |
|  |  | **RR**  **(95% CI)** | **P value** | **Adjusted RR**  **(95% CI)** | **P value** |
| **Age group** |  |  |  |  |  |
| <65 years | 6 | 0.76 (0.54, 1.06) |  | Reference |  |
| ≥65 years | 3 | 0.59 (0.39, 0.89) | 0.360 | 0.79 (0.32, 1.95) | 0.605 |
| **% of females** |  |  |  |  |  |
| <25 | 8 | 0.77 (0.59, 1.00) |  |  |  |
| ≥25 | 1 | 0.57 (0.36, 0.90) | 0.267 | Not included |  |
| **% of patients with diabetes** |  |  |  |  |  |
| <20 | 3 | 0.75 (0.43, 1.30) |  | Reference |  |
| ≥20 | 6 | 0.62 (0.43, 0.89) | 0.569 | 1.83 (0.66, 5.05) | 0.246 |
| **% of patients with hypertension** |  |  |  |  |  |
| <50 | 4 | 1.01 (0.56, 1.80) |  |  |  |
| ≥50 | 4 | 0.73 (0.56, 0.96) | 0.328 | Not included |  |
| **% receiving GP 2b/3a inhibitors** |  |  |  |  |  |
| <25 | 2 | 0.93 (0.67, 1.31) |  | Reference |  |
| ≥25 | 5 | 0.54 (0.38, 0.76) | 0.023 | 0.42 (0.20, 0.88) | 0.022 |

**SUPPLEMENTARY figure.1.** Funnel plot for primary outcome at 30 days.


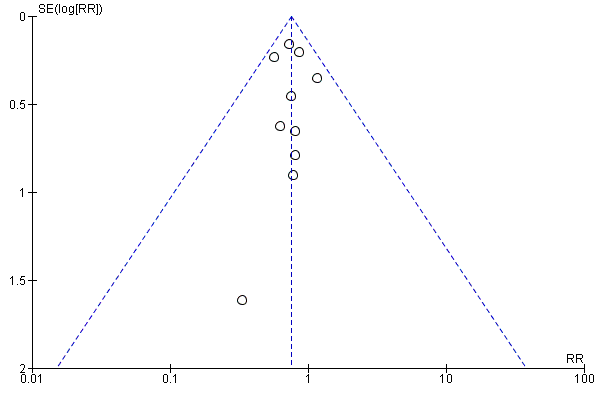


**\**

**Supplementary Figure. 2**

Sub-group analysis based on the nature of use of predominant anti-coagulant being bivalirudin.


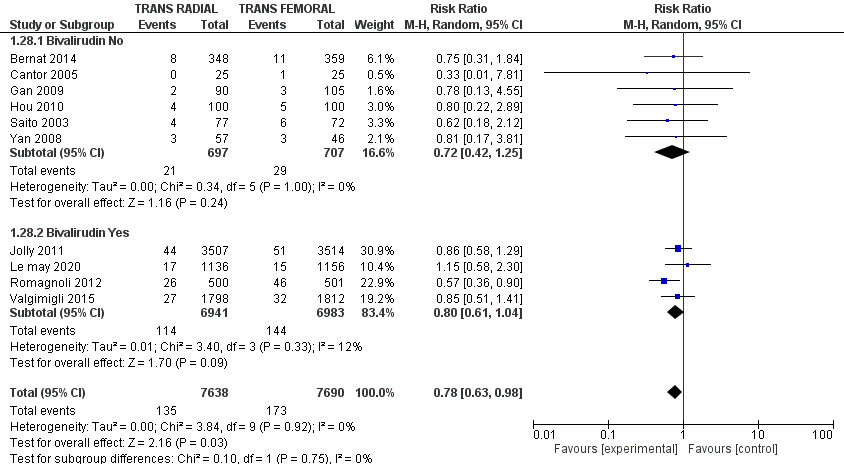


**Supplementary Figure. 3**

All-cause mortality at 30 days STEMI VS NSTEMI


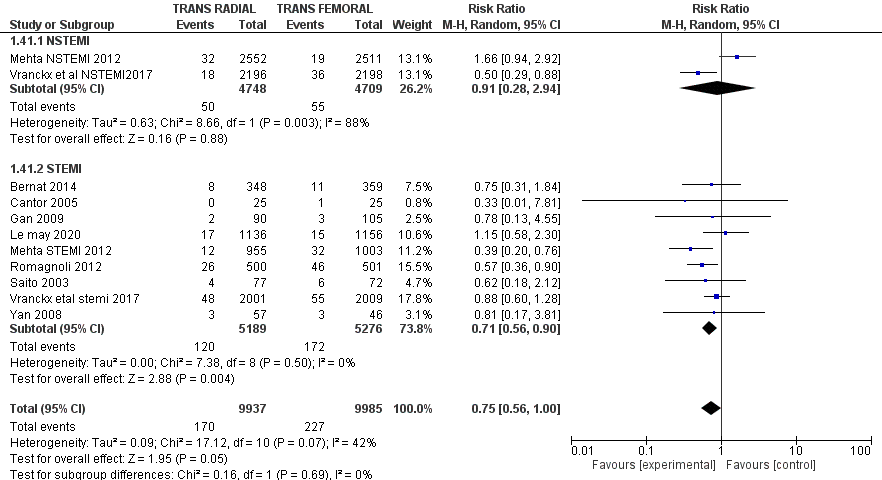


**Supplementary Figure. 4**

MACE


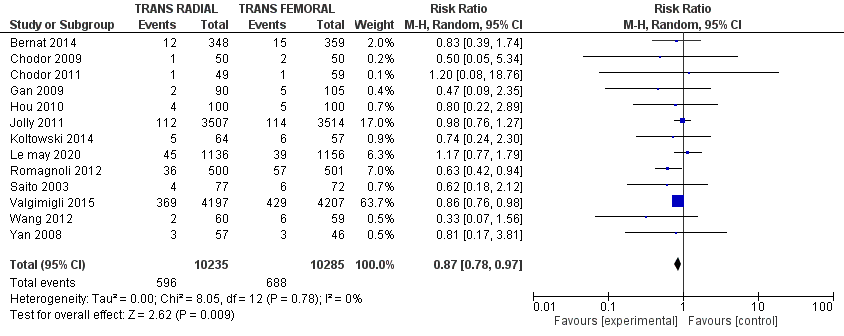


**Supplementary Figure. 5**

MACE high quality study


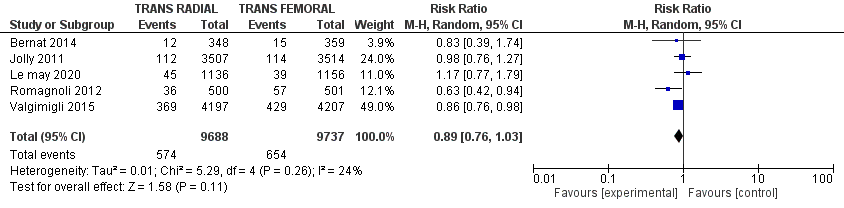


**Supplementary Figure.6**

MACE STEMI only


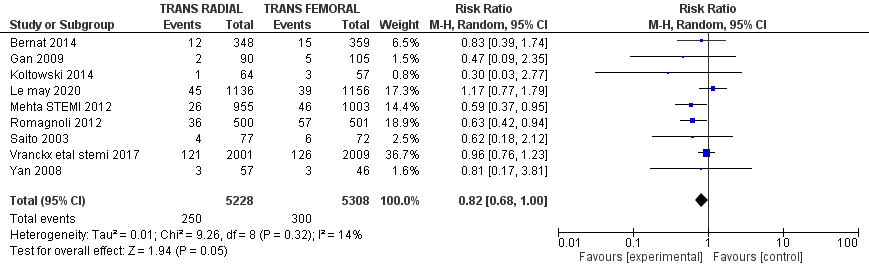


**Supplementary Figure.7**

MACE high quality studies


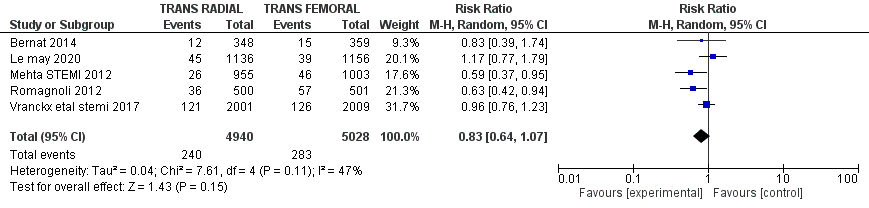


| **Supplementary Figure.8. (A-N). Anticipated and actual power of the meta-analysis for major outcomes**  **All-cause mortality at 30 days (all studies)** | |
| --- | --- |
| 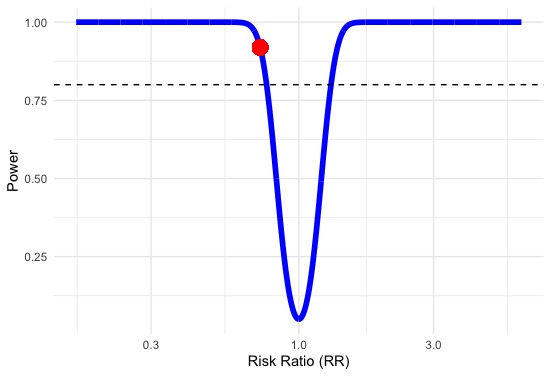   1. Anticipated power = 91.8% (k=10, n=100, RR=0.73) | 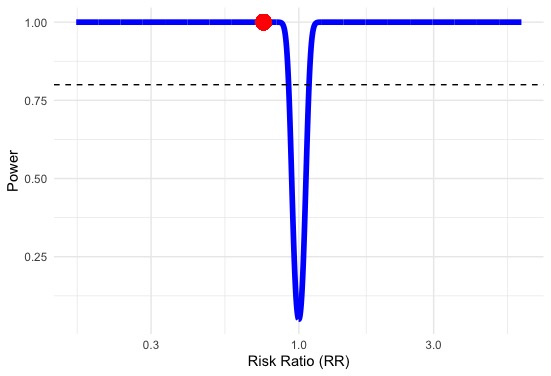   1. Actual power = 100% (k=11, n=900, RR=0.75) |
| **All-cause mortality at 30 days (high quality studies)** | |
| 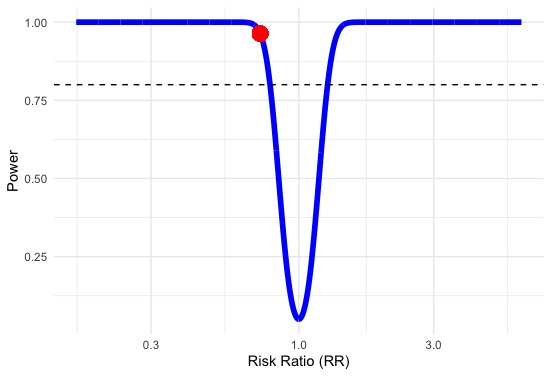   1. Anticipated power = 96.4% (k=5, n=250, RR=0.73) | 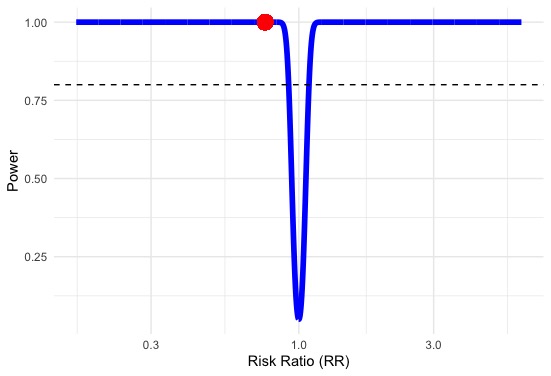   1. Actual power = 100% (k=5, n=2000, RR=0.76) |
| **MACE at 30 days (all studies)** | |
| 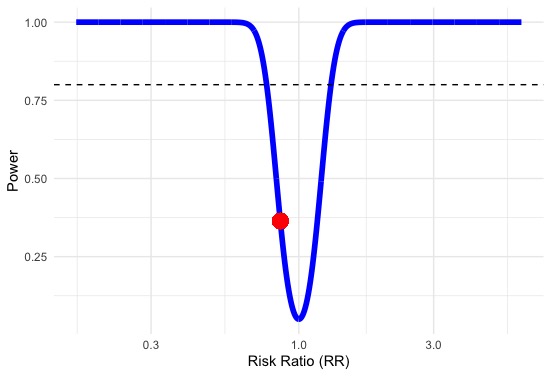   1. Anticipated power = 36.4% (k=10, n=100, RR=0.86) | 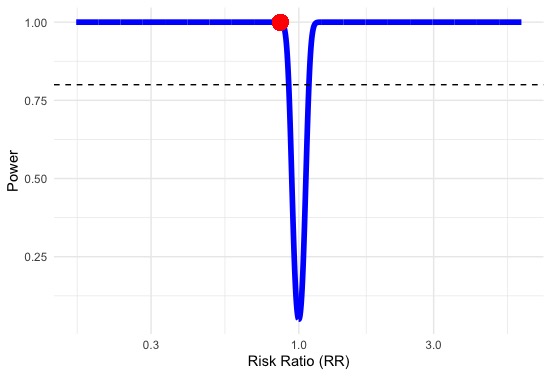   1. Anticipated power = 99.9% (k=13, n=780, RR=0.86) |
| **MACE at 30 days (high quality studies only)** | |
| 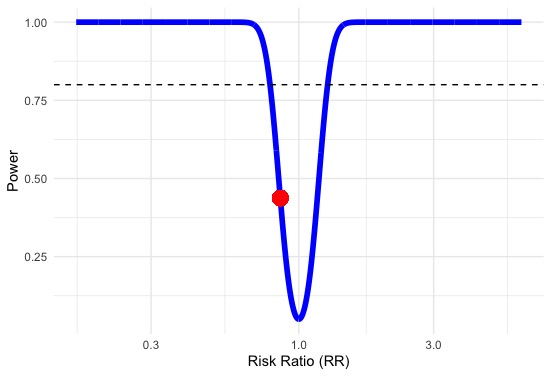   1. Anticipated power = 43.7% (k=5, n=250, RR=0.86) | 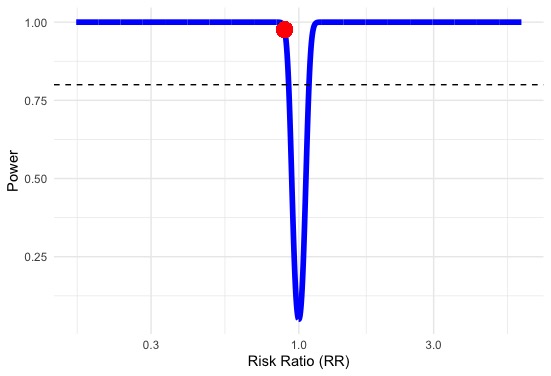   1. Actual power = 97.6% (k=5, n=2000, RR=0.89) |
| **All-cause mortality in STEMI (all studies)** | |
| 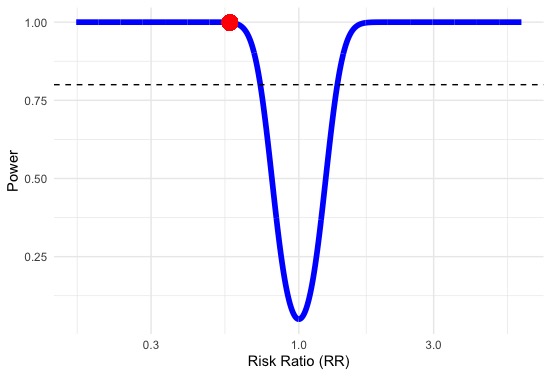   1. Anticipated power = 99.9% (k=7, n=100, RR=0.57) | 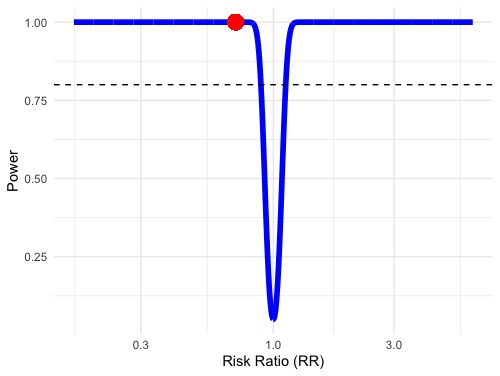   1. Actual power = 100% (k=9, n=600, RR=0.71) |
| **All-cause mortality in STEMI (high quality studies only)** | |
|  | 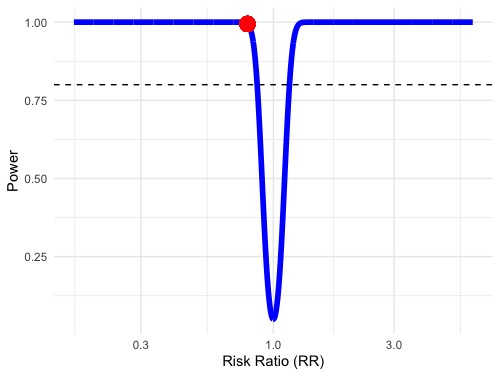   1. Actual power = 99.4% (k=4, n=1000, RR=0.79)* |

| **MACE in STEMI (all studies)** | |
| --- | --- |
| 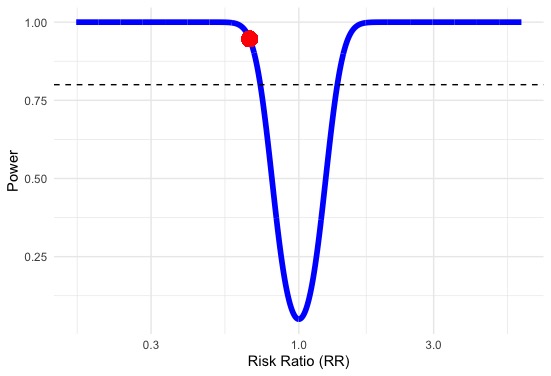   1. Anticipated power = 94.6% (k=7, n=100, RR=0.67) | 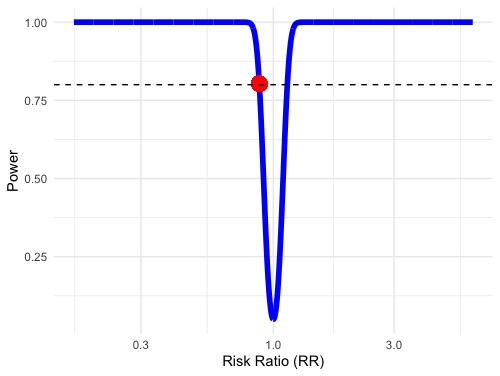   1. Actual power = 80.3% (k=8, n=530, RR=0.88) |
| **MACE in STEMI (high quality studies only)** | |
|  | 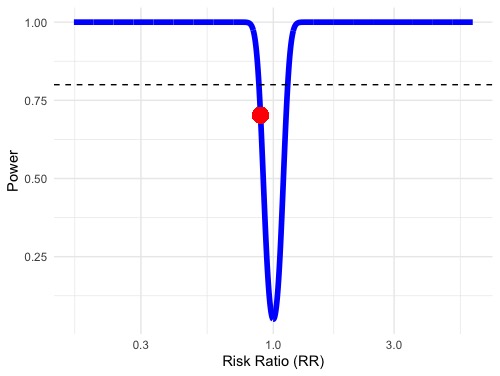   1. Actual power = 70.2% (k=4, n=1000, RR=0.89) |
|  |  |

Note: For all calculations, significance level was kept at 5% and heterogeneity was kept as low. For * heterogeneity was kept as moderate. Dashed line in black represents the 80% power threshold. **MACE**-Major adverse cardiac events

**Supplementary Figure.8. (A-N). Anticipated and actual power of the meta-analysis for major outcomes**

For primary outcome at 30 days, both anticipated and actual power was very high (>90%) regardless of the quality of studies(A-D). For MACE at 30 days, the actual power was much higher (>95%) compared to the anticipated power of <45%, regardless of the quality of studies(E-H).Among STEMI, a similar pattern was observed with >99% power for primary outcome at 30 days, regardless of the quality of studies included (I-K). For MACE among STEMI, the actual power for all included studies (80.3%) and only high quality studies (70.2%) were much lower than the anticipated power of 94.6% (L-N).

**Supplementary Figure 9. Bubble plot showing the relationship between treatment effect and percentage of patients receiving GP 2b/3a inhibitors in patients with STEMI**


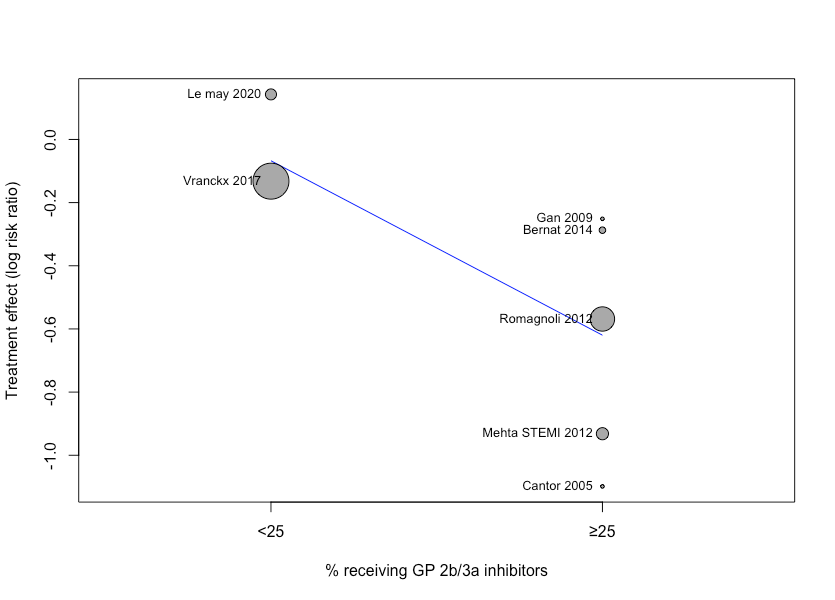


**Supplementary Figure.10**

**BARC 3-5 bleeding**


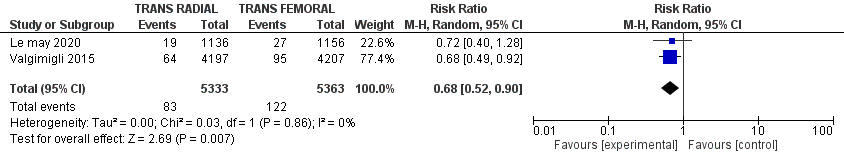


**Supplementary Figure.11**

Minor bleeding


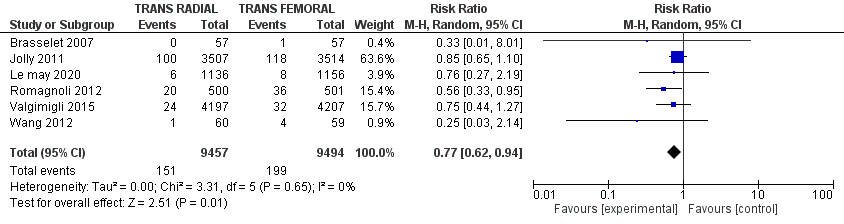


**Supplementary Figure.12**

Severe bleeding requiring blood transfusions


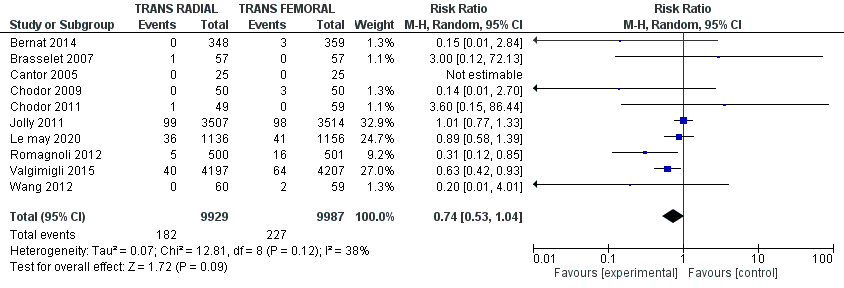


**Supplementary Figure.13**

In-hospital mortality


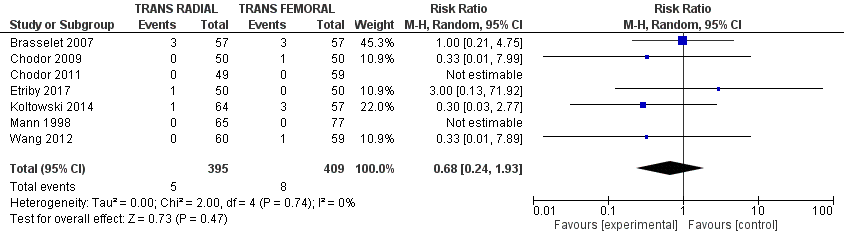


**Supplementary Figure 14**

Access-site crossovers


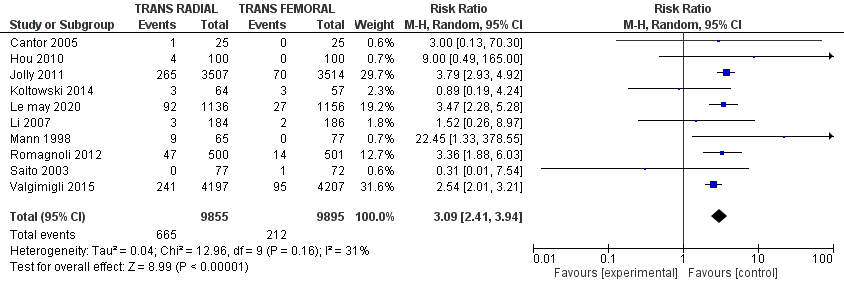


**Supplementary figure.15**

Contrast volume

**
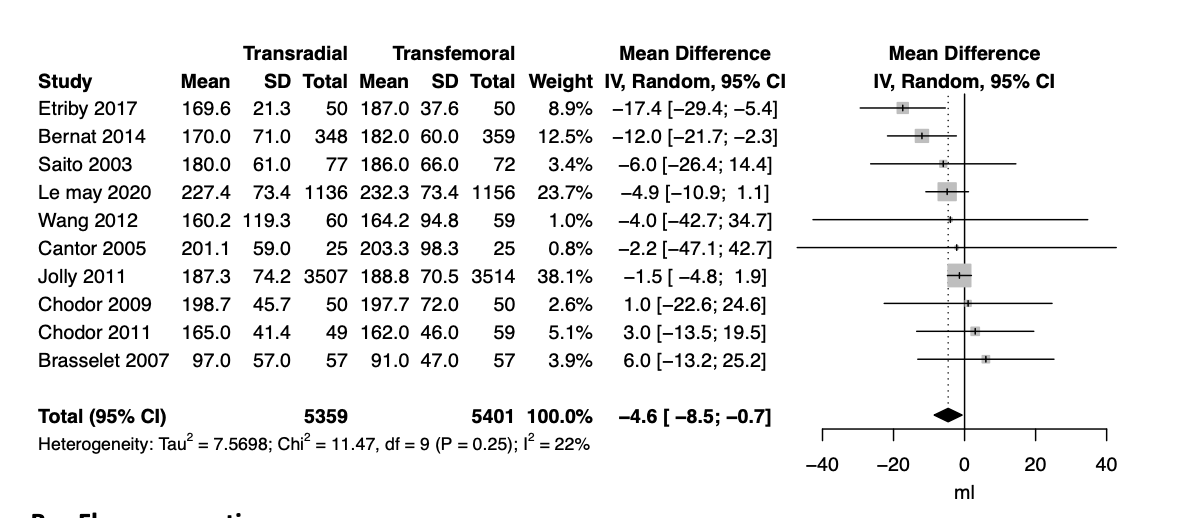
**

**Supplementary Figure.16**

Fluoroscopy time

**
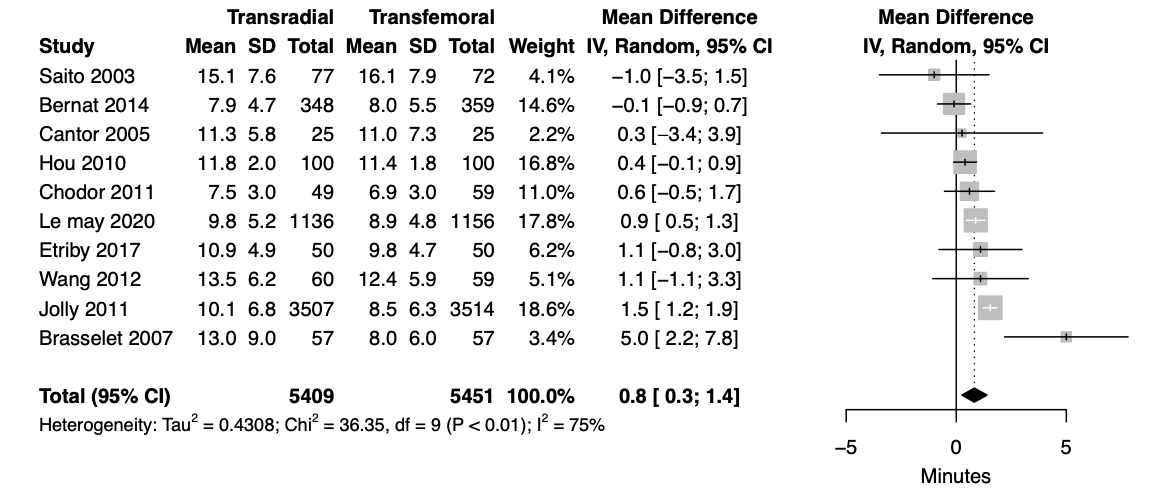
**

**Supplementary Figure.17**

Procedure time

**
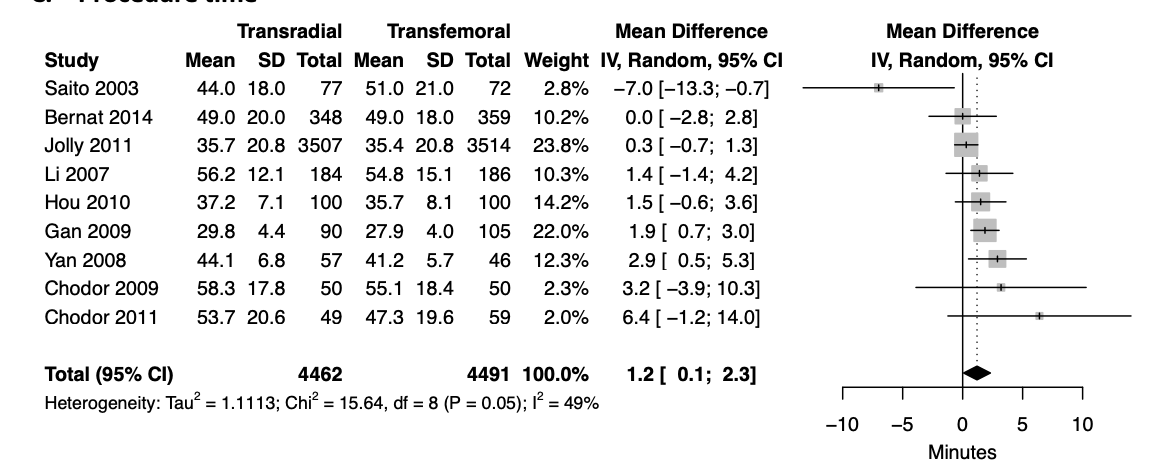
**

**Supplementary Figure.18**

Arrival at PCI to FBI

**
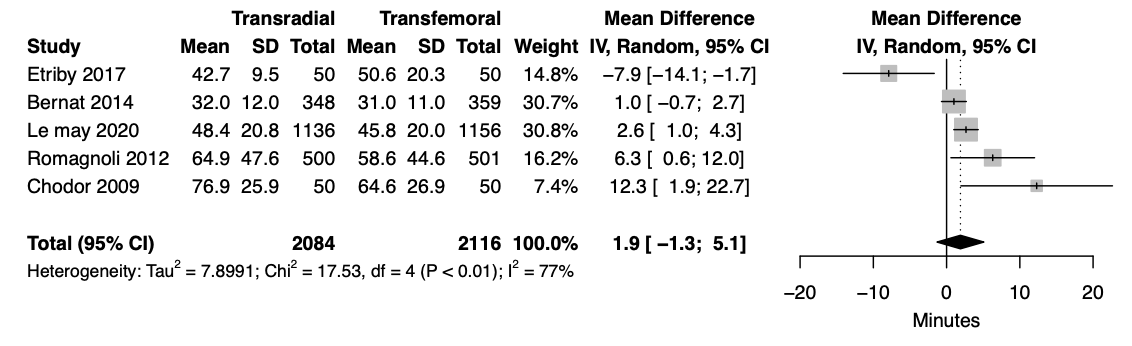
**

**FBI:** First balloon inflation

**Supplementary Figure 19**

Study defined Acute Kidney Injury

**
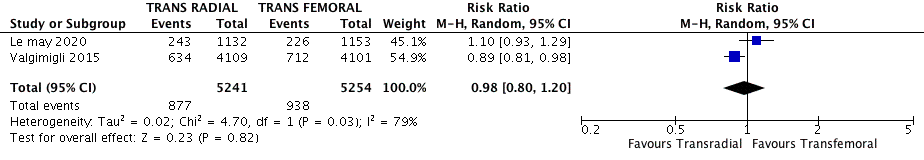
**

**Supplementary Figure 20**

Mean difference in creatinine (mg%)**
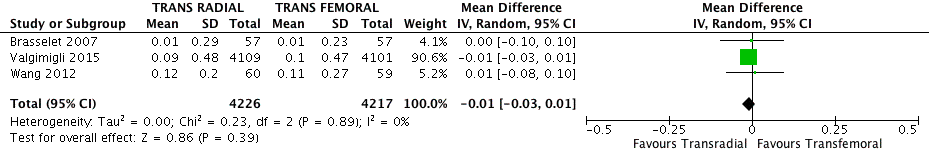
**
